# Stochastic molecular model of enzymatic hydrolysis of cellulose for ethanol production

**DOI:** 10.1186/1754-6834-6-63

**Published:** 2013-05-02

**Authors:** Deepak Kumar, Ganti S Murthy

**Affiliations:** 1 Biological and Ecological Engineering, Oregon State University, Corvallis, USA

**Keywords:** Cellulose hydrolysis, Bioethanol, Hydrolysis modeling, Cellulase, Synergism, Exo-cellulase, Endo-cellulase

## Abstract

**Background:**

During cellulosic ethanol production, cellulose hydrolysis is achieved by synergistic action of cellulase enzyme complex consisting of multiple enzymes with different mode of actions. Enzymatic hydrolysis of cellulose is one of the bottlenecks in the commercialization of the process due to low hydrolysis rates and high cost of enzymes. A robust hydrolysis model that can predict hydrolysis profile under various scenarios can act as an important forecasting tool to improve the hydrolysis process. However, multiple factors affecting hydrolysis: cellulose structure and complex enzyme-substrate interactions during hydrolysis make it diffucult to develop mathematical kinetic models that can simulate hydrolysis in presence of multiple enzymes with high fidelity. In this study, a comprehensive hydrolysis model based on stochastic molecular modeling approch in which each hydrolysis event is translated into a discrete event is presented. The model captures the structural features of cellulose, enzyme properties (mode of actions, synergism, inhibition), and most importantly dynamic morphological changes in the substrate that directly affect the enzyme-substrate interactions during hydrolysis.

**Results:**

Cellulose was modeled as a group of microfibrils consisting of elementary fibrils bundles, where each elementary fibril was represented as a three dimensional matrix of glucose molecules. Hydrolysis of cellulose was simulated based on Monte Carlo simulation technique. Cellulose hydrolysis results predicted by model simulations agree well with the experimental data from literature. Coefficients of determination for model predictions and experimental values were in the range of 0.75 to 0.96 for Avicel hydrolysis by CBH I action. Model was able to simulate the synergistic action of multiple enzymes during hydrolysis. The model simulations captured the important experimental observations: effect of structural properties, enzyme inhibition and enzyme loadings on the hydrolysis and degree of synergism among enzymes.

**Conclusions:**

The model was effective in capturing the dynamic behavior of cellulose hydrolysis during action of individual as well as multiple cellulases. Simulations were in qualitative and quantitative agreement with experimental data. Several experimentally observed phenomena were simulated without the need for any additional assumptions or parameter changes and confirmed the validity of using the stochastic molecular modeling approach to quantitatively and qualitatively describe the cellulose hydrolysis.

## Background

Lignocellulosic biomass is a complex matrix of three biopolymers: cellulose (20-50%), hemicellulose (15-35%) and lignin (5-30%) [1-3]. Conversion of renewable lignocellulosic biomass into biofuels is at the heart of advanced biofuels production. Two important biochemical approaches to accomplish this involve enzymes and/or cellulolytic microorganisms. First approach involves pretreatment of lignocellulosic feedstocks [4-6], followed by hydrolysis and subsequent/simultaneous fermentation by yeasts [7,8]. Second approach involves us of cellulolytic microbes with consolidated bioprocessing (CBP) capabilities that hydrolyze cellulose without external enzyme addition [9]. The first approach represents state of the art in cellulosic ethanol and is the system of choice for near term (5–10 years) commercialization that is being tested at pilot and industrial scales.

### Cellulose and diversity of cellulases in nature

Hydrolysis of cellulose and hemicellulose into sugar monomers is a critical step in the biochemical conversion of cellulose into ethanol. Cellulose is the most abundant biopolymer on earth with about 100 billion tons produced by terrestrial plants every year [10]. Cellulose is made of glucose units linked together by β-1,4 glycosidic bonds. Due to extensive hydrogen bonding, cellulose chains form a recalcitrant crystalline structure that has a half-life of several million years at neutral pH and ambient temperatures in the absence of enzymes [10].

However, abundance of cellulose in diverse natural substrates has led to evolution of diverse cellulolytic organisms capable of degrading cellulose [11-14]. There are three main mechanisms of cellulose degradation found among most cellulolytic organisms [10]. First mechanism which is used by many aerobic organisms is the extracellular secretion of free cellulases. *Trichoderma reesei (T. reesei)* is a well-studied fungus that employs this mechanism. Second mechanism employed by most anaerobic bacteria such as *Clostridium thermocellum* is the use of large multienzyme complexes called cellulosomes. Third strategy, comparable in effectiveness to above strategies, employed by organisms such as *F. succinogens* (anaerobic rumen bacteria) and *Cytophaga hutchinsonii* (aerobic soil bacterium) is presently not well understood. These organisms do not produce cellulosomes or secrete extracellular enzymes and remain tightly bound to the cellulose substrate [15].

**Figure 1:**
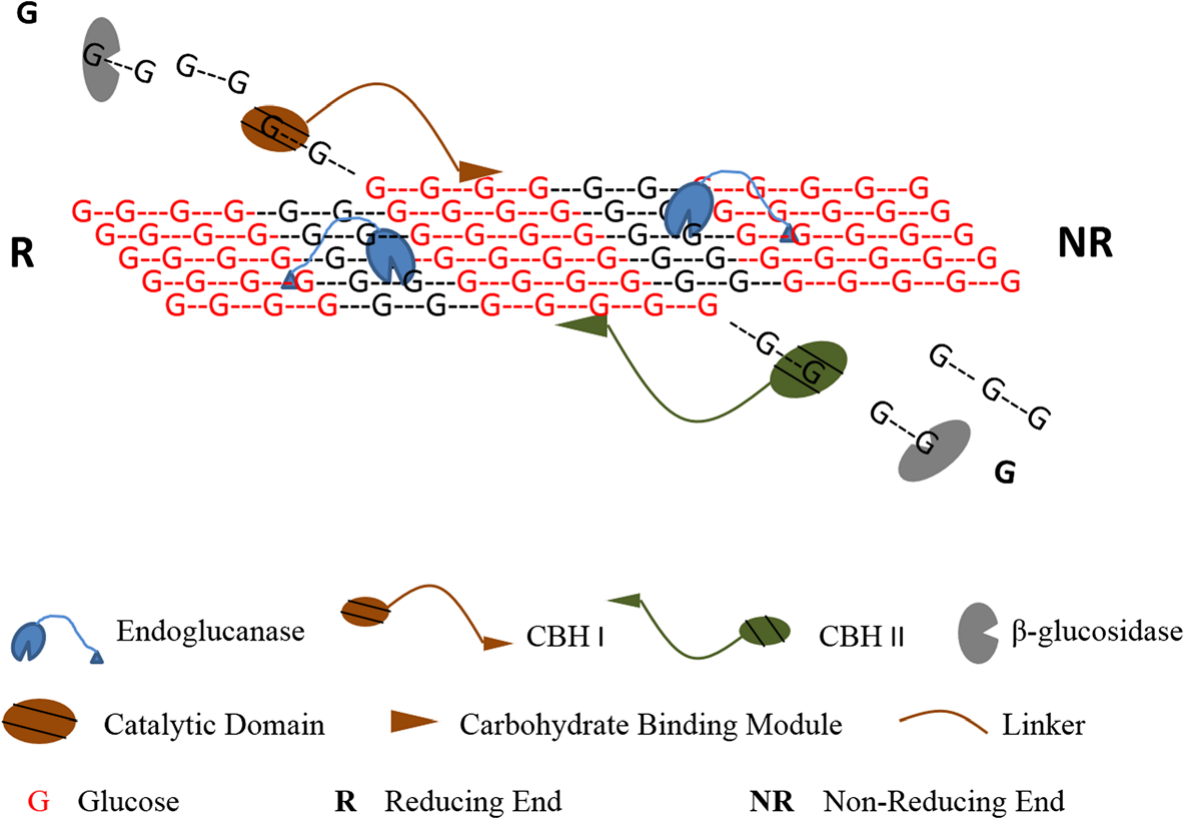
**Action of various cellulases enzymes on surface layer of cellulose.** Figure illustrates the action of enzymes from three main classes on cellulose. Glucose molecules in red color represents crystalline region and glucose molecules in black color are in amorphous region.

Numerous cellulases produced in nature can be classified into three main classes of enzymes: Endoglucanses (EG), exoglucanases (cellobiohydrolase I and II) and β-glucosidases [Please see [1] for an excellent review]. While the mode of action of all these enzymes is different, they exhibit synergism which results in efficient cellulose degradation (Figure 1). Endocellulases bind randomly along a glucose chain and hydrolyse one/few accessible bonds. Exoglucanases (cellobiohydrolases, CBH) are divided into two categories: Type I attack from the reducing end of the chain while the enzymes of type II attack the non-reducing end of the glucose chain producing a cellobiose molecule (a dimer of glucose). Exoglucanases in general are processive, i.e. the enzymes remain bound to the glucose chain after cleaving a cellobiose molecule and will continue to cleave cellobiose units until a minimum chain length is reached [13]. Most Endoglucanases on the other hand are non-processive; however a new class of processive endoglucanases has been identified in bacteria [15]. In addition to the endo/exoglucanases enzymes, all cellulolytic organisms also produce free/membrane bound β-glucosidases which act on cellobiose/cellodextrins to produce glucose. Finally in addition to these enzymes, a large family of glycoside hydrolase family 61 (GH61) has recently been identified [16]. The GH61 family of proteins although lacking measurable hydrolytic activity in standalone experiments had a significant synergistic effect in enhancing the efficacy of other cellulolytic enzymes in presence of divalent cations. Interestingly, similar accessory proteins were also discovered to increase efficiency of enzymes from chitinolytic organisms [17].

Aerobic organisms (e.g. *T. reesei*) utilize cellulose by secreting free cellulase enzymes, also known as 'enzyme system', extracellularly [1,10,18-22].

Enzymatic hydrolysis process can be divided into two stages: In the first stage, long chains are hydrolyzed to form soluble oligomers, and soluble oligomers are in turn hydrolyzed to sugar monomers during the second stage of hydrolysis. First stage of hydrolysis is considered as a rate limiting step in the hydrolysis process [1,20]. Several experimental studies have concluded that physical properties of cellulose such as crystallinity, degree of polymerization (DP) and accessible surface area, are some of the major factors responsible for controlling hydrolysis rate due to the effect on enzyme binding and substrate accessibility to the cellulase enzymes [23-25]. Crystallinity is a key factor affecting the hydrolysis of cellulose as the glycosidic bonds in crystalline regions are difficult to hydrolyze compared to those in the amorphous regions [23,25].

Enzymatic cellulose hydrolysis process is one of the bottlenecks and key cost center (enzymes costs up to $1/gal ethanol) in the commercialization of process of cellulosic ethanol production [22,26-30]. However, there is potential for cost reduction by improving the understanding of the process, testing enzymes and various substrates under different conditions to determine optimum hydrolysis conditions. Since conducting hydrolysis experiments is time consuming and labor intensive, a comprehensive hydrolysis model that can predict accurate hydrolysis profile of cellulose under various scenarios (substrate, enzyme, hydrolysis conditions) can be used as an important forecasting tool to understand and improve the hydrolysis process [22,26].

### Cellulose hydrolysis modeling

There are three principal approaches to model cellulose hydrolysis by non-complexed systems [20,27]. First approach is to fit experimental data to linear/nonlinear regression models which can be simple to construct but require large sets of experimental data.

Second approach involves formulation of mechanistic models which attempt to model some of the underlying phenomenon with simplifying assumptions [20,22,27,31-37]. Rate expressions are generally described using Michaelis-Menten type enzyme kinetics with/without incorporating the effects of enzyme adsorption, temperature, pH, substrate and product inhibition. The model structure results in a set of ordinary differential equations (ODE) and model parameters are often determined by fitting model predictions to the experimental data. Substrate and product inhibition, lignin inhibition effect, cellulose accessibility and reactivity effects have been considered in some of these models. These models are reasonably accurate in predicting the experimental trends of sugar production and are currently most widely used in literature to predict cellulose hydrolysis [20,27]. While a more general set of conditions can be simulated by incorporating additional model terms this often results in the loss of physical significance of the model terms and leads to overparameterization issues. Due to these limitations, a new parameter set must be identified whenever the substrate, pretreatment conditions, enzymes and/or process conditions are changed. Another limitation of this modeling approach is that synergistic interaction of various enzymes is difficult to determine and model accurately. Some of the models in literature do account for endo/exo and exo/exo synergistic interactions [33]. However due to the reliance on lumped terms and inadequate detail in describing cellulase-cellulose interactions, their predictive abilities are limited [34].

Most of these kinetic models can not consider dynamic change in properties of substrate (accessibility of enzymes) that directly affect the enzyme-substrate interactions and rate of hydrolysis in detail. At most some of the models have considered changes in accessibility based on ratio of active/less active cellulose, amorphous/crystalline cellulose and surface area. However it is very difficult to consider accessibility changes for individual enzymes depending upon their mode of action.

Stochastic molecular modeling (SMM) of the hydrolysis in which each hydrolysis event in translated into a discrete event is another approach that can be used for modeling cellulose hydrolysis that can capture dynamic enzyme-substrate intearction during hydrolysis. This approach relying on modeling enzymatic hydrolysis process at molecular and enzymatic levels has been successful in describing starch hydrolysis [38-42]. These studies concluded that stochastic molecular modeling technique can be used to predict hydrolysis profile and addressing the limitations of kinetic models (e.g. mathematical complexities, large number of parameter estimations, change in parameters with change enzyme or hydrolysis conditions etc.). One of the main advantages of the SMM approach is that structural characteristics and enzyme characteristics can be separately determined and incorporated into the model. Some other specific advantages of SMM models are:

1. Changes in substrate property, enzyme characteristics can be incorporated without the need for additional experimentation.

2. Concentrations of many oligomers can be tracked without increasing the complexity of model.

3. Chain distribution (number of chains with different chain lengths) during hydrolysis can be easily determined using SMM approach, which can provide better understanding of action mechanism and behavior of enzymes during hydrolysis.

4. Changes in structural properties such as number of chain ends, average degree of polymerization and crystallinity over time can be followed during hydrolysis.

The basic requirement of this approach is detailed and accurate description of substrate properties and enzymes action in the model. Although SMM approach is computationally intensive, it is more realistic and can easily incorporate the changes in conditions (substrate, enzyme or reaction conditions). However, potential of this technique has not been explored in detail for cellulose hydrolysis.

The first reported model for hydrolysis of insoluble polysaccharides using stochastic modeling approach was developed by Fenske et al. [43]. This was a limited SMM model as it did not capture the actual structural properties of cellulose (e.g. crystallinity, DP, fibril structure) and multi enzyme dynamics. Hydrolysis was performed on a two dimensional matrix representing a single surface of cellulose with short chain length of 20. It was a theoretical study and results were not validated with experimental data. More recently, similar approach was used by Asztalos et al. [44] to model cellulose hydrolysis which had reasonable accuracy in predicting the hydrolysis trends for endoglucanse and CBH enzymes. Dynamic enzyme-substrate interactions were captured to some extent in the model. However the model did not include some important structural features of cellulose and had a limited usability. It was a two dimensional model in which all glucose chains were accessible to all enzymes, which in not the case in actual process. Degree of crystallinity was not considered in the cellulose structure, and consequently, activity difference of enzymes in amorphous and crystalline regions found to be significant by many researchers was not incorporated into the model [1,20,27]. Inhibition by cellobiose or glucose was not accounted into the model, which is very important parameter during cellulose hydrolysis. Therefore, despite these relatively recent advances, there is no SMM model to date that considers complex enzyme-substrate interactions. The objective of this study was to develop a detailed SMM model that can predict hydrolysis profiles of cellulose with high accuracy by capturing the complexities of cellulose structure and hydrolysis mechanism. The aim was to develop a general hydrolysis model that can be used for various conditions (different substrates, enzymes, hydrolysis conditions) considering structural properties of feedstock (crystallinity, degree of polymerization, accessibility), enzyme properties (mode of actions, synergism and inhibition) and most importantly dynamic changes in these properties during hydrolysis.

## Results and discussion

### Model Validation

#### Validation data set 1: Hydrolysis of Avicel

**Figure 2:**
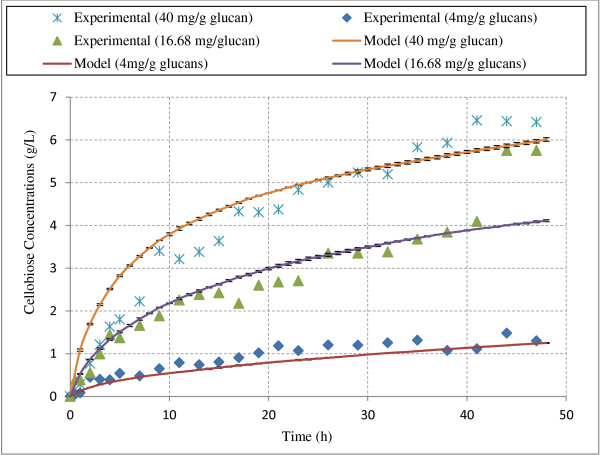
**Cellobiose production during hydrolysis of Avicel (25 g/L) at various loadings of CBH I.** The figure compares model simulations with experimental data from hydrolysis of Avicel by CBH I. The data points are from Bezerra et al. [45-47] and the lines are from the model predictions. “mg/g glucans” indicates the loading of CBH I enzyme in mg per gram of glucans.

**Figure 3:**
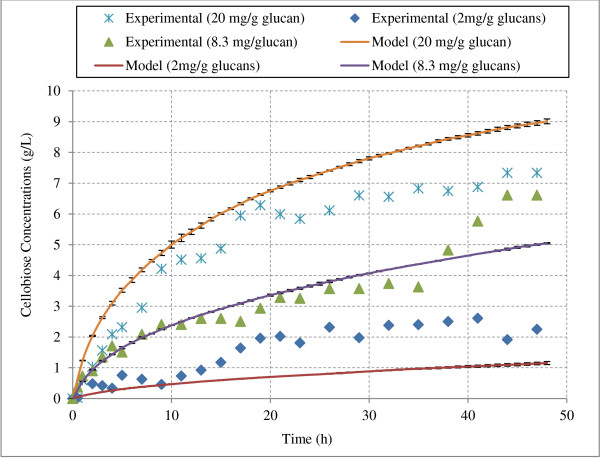
**Cellobiose production during hydrolysis of Avicel (50 g/L) at various loadings of CBH I.** The figure compares model simulations with experimental data from hydrolysis of Avicel by CBH I. The data points are from Bezerra et al. [45-47] and the lines are from the model predictions. “mg/g glucans” indicates the loading of CBH I enzyme in mg per gram of glucans.

For model validation, amounts of cellobiose production during hydrolysis of Avicel by CBHI at various experimental conditions were compared to experimental data [45-47]. Using the model described above, hydrolysis simulations were performed for the exact experimental conditions on simulated Avicel structure. Cellobiose production from Avicel hydrolysis based on experimental and model simulation data were in qualitative and quantitative agreement at 25 and 50 g/L Avicel loadings (Figures 2 and 3).

To check the repeatability of model results, these simulations were performed in triplicate for each condition and average results along with error bars (standard deviations) have been presented in Figures 2 and 3. Standard deviations found from three simulations were very small in each case (data for all three simulations is provided in section A5 of the supplementary material). Coefficient of determination (R^2^, defined as the square of the Pearson product–moment correlation coefficient) was calculated to determine the statistical agreement of model simulation data with experimental data. R^2^ value of 1 indicates perfect model validation with experimental values. The R^2^ values were found to be greater than 0.75 in all model simulations (Table 1) indicating a good agreement with the experimental values. Quantitative match of model simulations with experimental data further indicates that this model was able to capture cellobiose inhibition effect under various hydrolysis conditions (different enzyme/substrate ratios).

**Table 1:** Correlation between experimental and model simulation data for Avicel hydrolysis by CBH I action at different conditions

**25 g/L Avicel**		**50 g/L Avicel**	
**Enzyme loading (mg/g glucans)**	**R**^**2 **^**Value**	**Enzyme loading (mg/g glucans)**	**R**^**2 **^**Value**
4	0.91	2	0.80
16.68	0.75	8.3	0.80
40	0.80	20	0.96

#### Validation data set 2: hydrolysis of Avicel

**Figure 4:**
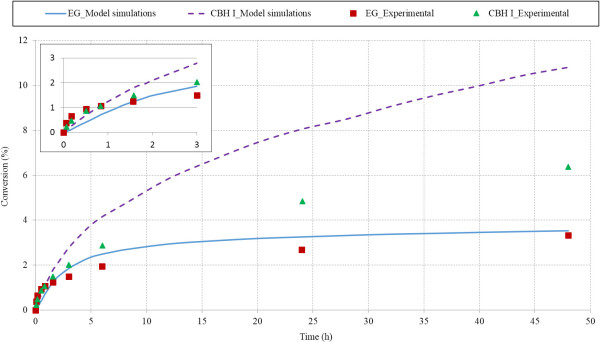
**Cellulose conversion during hydrolysis of Avicel (10 g/L) by action of EGII (8 mg/g glucan) and CBHI (10 mg/g glucan).** The figure compares model simulations with experimental data from hydrolysis of Avicel by endoglucanases (EG) and cellobiohydrolase (CBH I). The data points are from Medve et al. [48] and the curves are from the model predictions. The insert in the figure illustrates data points and model simulations for initial 3 h of hydrolysis.

Model results were further tested by comparing results with experimental data from Medve et al. [48]. Model data fitted well with experimental data for EGII (R^2^ = 0.90). Cellulose conversion data from model simulations for CBH I showed good match with experimental values during initial hydrolysis period (initial 3 hours, Figure 4 insert) (R^2^ = 0.91), however model simulations did not match experimental results quantitatively at later stages of hydrolysis. Cellulose conversion by CBHI action after 48 h of hydrolysis was experimentally determined to be 6.5%, whereas model predictions for final conversion was 10.8%.

**Figure 5:**
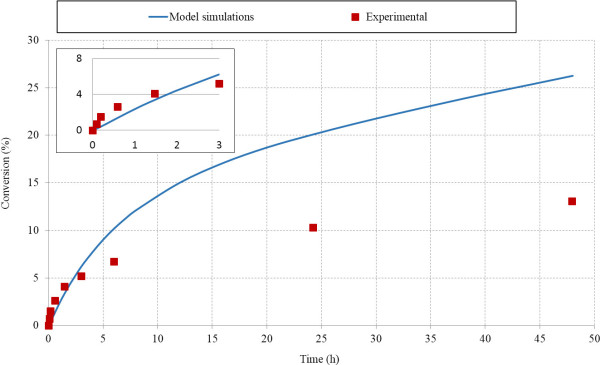
**Cellulose conversion during hydrolysis of Avicel (10 g/L) by combined action of EGII (8 mg/g glucan) and CBHI (10 mg/g glucan).** The figure compares model simulations with experimental data from hydrolysis of Avicel by synergistic action of endoglucanases (EG) and cellobiohydrolase (CBH I). The data points are from Medve et al. [48] and the lines are from the model predictions. The insert in the figure illustrates data points and model simulations for initial 3 h of hydrolysis.

During Avicel hydrolysis using a mixture of EG and CBHI, model simulations match experimental results qualitatively and quantitatively in the initial stage of hydrolysis (Figure 5). However, the model results were only qualitatively similar to the experimental results and there was divergence in the last two experimental points.

There are several possible reasons for this divergence in the later stages of hydrolysis. Commercial Avicel substrates obtained from different sources vary in their physical properties, whereas we used average values of DP and crystallinity index for structure simulations. Another important contributor to the difference could be differences in enzyme activities. A great variability has been found among of activities of pure enzymes, depending upon degree of purification [20]. Activity used in the model simulations were used from other literature studies, which might be different from the activity of CBH I purified in these experiments.

### Enzymatic hydrolysis of cellulose by individual enzymes

**Figure 6:**
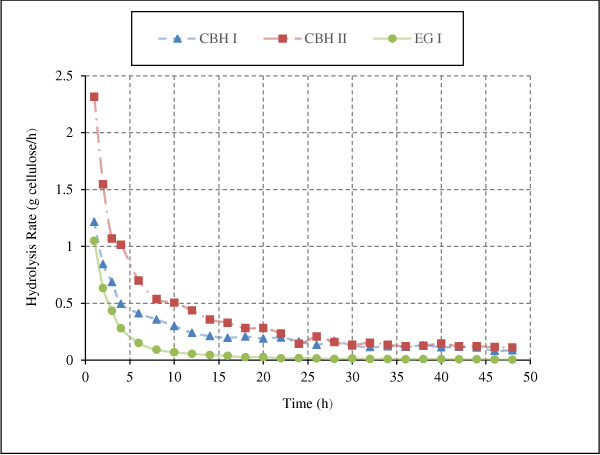
**Model predictions of hydrolysis rate by action of individual enzymes on Avicel.** Figure depicts the rate of hydrolysis of Avicel by action of individual enzymes (Avicel, 100 g/L; all enzymes, 10 mg/g glucans).

**Figure 7:**
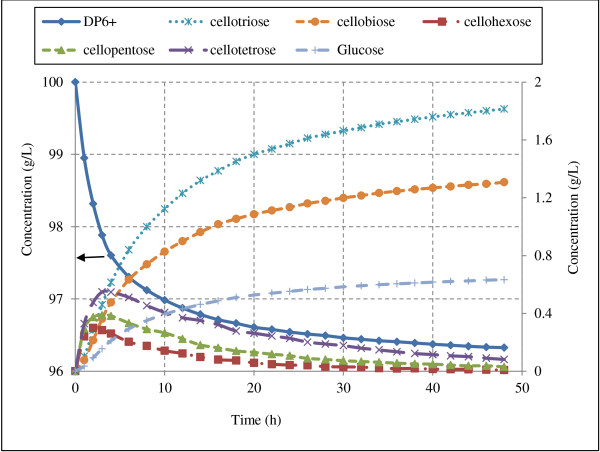
**Model predictions of sugar concentrations during hydrolysis of Avicel by endoglucanase.** Figure illustrates the sugars production profile during Avicel hydrolysis by endoglucanases action only (Avicel, 100 g/L; EGI, 10 mg/g glucans) [DP6+ on primary axis to the left and rest on secondary axis to the right].

The hydrolysis model was simulated for microcrystalline cellulose (Avicel) hydrolysis by individual enzymes. Structure of Avicel was simulated as described in the methods section. Model simulations were performed assuming 100 g/L substrate concentration at enzyme loadings of 10 mg/g glucans for 48 hours of hydrolysis. Cellulose hydrolysis rates due to individual enzymes (EGI, CBH I and CBH II) is presented in Figure 6. The production profiles of soluble sugars and cellulose (DP6+) conversion during hydrolysis of cellulose by endoglucanases are shown in Figure 7.

For all three enzymes the hydrolysis rates decrease rapidly after few initial hours of hydrolysis and then become nearly constant (Figure 6). The decrease in hydrolysis rate with cellulose conversion is a widely observed phenomenon that is expected due to morphological changes in the structure of cellulose such as decrease in glucose chains on microfibril surface. These morphological changes limit the accessibility of cellulase enzymes to glucose chains and results in rapid decline in hydrolysis rate [20,36].

During hydrolysis of cellulose by CBH I and CBH II, cellobiose is the only major product formed, which is a strong inhibitor of these enzymes [1,20,27] and limits the hydrolysis rate. Concentration of cellotriose, cellobiose and glucose were higher than that of cellotetrose, cellopentose and cellohexose after 48 h hydrolysis of Avicel by EGI (Figure 7). Concentrations of cellotetrose, cellopentose and cellohexose increase during initial few hours (3–4 h) of hydrolysis and then decrease in the latter stages of hydrolysis. This trend is expected since during initial phase of hydrolysis surface glucose chains are easily accessible and endoglucanases acts randomly on them to producing short chains. However, with the progress of hydrolysis accessibility of enzymes to the glucose chains decreases. The enzymes start acting on soluble sugars and reduce their overall concentrations after an initial increase. Concentration of cellobiose and cellotriose do not decrease as EG I was assumed to act only on oligomers with DP>4. In case of hydrolysis by CBH I and CBH II, all soluble sugars except cellobiose were produced in negligible amounts (less than 0.01 g/L, data not reported).

### Effect of structural properties of cellulose

**Figure 8:**
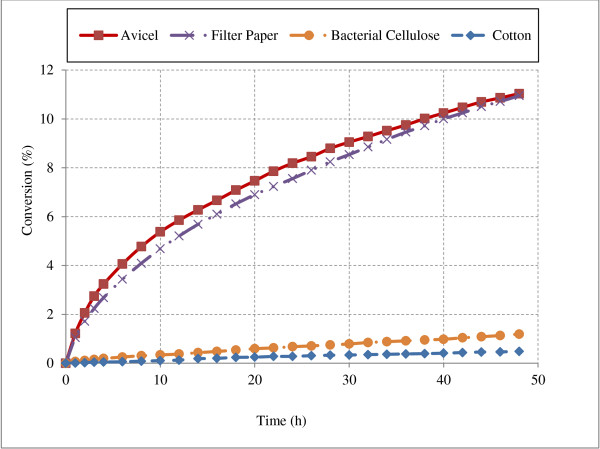
**Hydrolysis of various cellulose substrates by CBH I enzyme.** Figure illustrates the cellulose conversion profile during hydrolysis of various cellulose substrates (100 g/L) by action of CBH I enzyme (10 mg/g glucans).

Hydrolysis of cellulose is highly dependent on structural properties of cellulose. For example, cellobiohydrolases (CBH I and CBH II) act on reducing/non-reducing ends of glucose chains, so length of the glucose chains in the substrate, which represent the fraction of reducing/non-reducing ends compared to total glucose molecules, directly affect the hydrolysis action of these enzymes. For example, the percentage of reducing/non-reducing ends to total glucose molecules for Avicel with average chain DP of 300 is 0.33% compared to 0.05% for bacterial cellulose with average DP of 2000 [20,33]. Therefore it would be expected that CBH I and CBH II would hydrolyze pure Avicel to a greater extent compared to bacterial cellulose as there are many more reducing/non-reducing ends. To determine the model accuracy in predicting this trend, action of CBH I was simulated on various cellulose substrates shown in Table 2 and results are shown in Figure 8.

**Table 2:** Key Properties of simulated model cellulosic substrates

**Model Substrate**	**Degree of polymerization (DP)**	**Crystallinity index (CrI)**	**Number of microfibrils simulated (Total glucose molecules simulated)**
Avicel	250-300	0.5-0.6	5 (1090224)
Filter Paper	700-800	0.4-0.5	4 (2684088)
Bacterial Cellulose (BC)	1800-2000	0.85-0.95	2 (2999880)
Cotton	2500-3000	0.85-0.95	2 (3345264)

It can be observed from Figure 8 that model simulations agreed with the expected results and captured the inverse relationship between substrate DP and hydrolysis of cellulose. Cellulose conversion after 48 h hydrolysis of high DP substrates: bacterial cellulose (DP 1800–2000) and cotton (DP 2500–3000), which have low availability of chain ends for CBH attack, was only 10.8 and 4.4% relative to that from low DP substrate, Avicel (DP 250–300) hydrolysis. Similar results have been reported in literature from both experimental as well as modeling studies [33,49]. Although it appears that cellulose conversion was almost similar for Avicel and filter paper, rate of hydrolysis was lower in case of filter paper. After few hours of hydrolysis, cellobiose inhibition becomes dominant and limits the hydrolysis.

Endoglucanases activity is affected by degree of crystallinity. Regions that are highly crystalline are less susceptible to hydrolysis compared to amorphous regions because of low accessibility of enzymes in these regions [25]. This behavior was also captured by model simulations as cellulose conversion by action of endoglucanases on cotton (highly crystalline cellulose, CrI 0.85-0.95) was found 42.8% lower than that of Avicel (semi-crystalline cellulose, CrI 0.5-0.6) (Figure A6, section A6 in the Additional file 1).

### Synergism

Cooperative action of different enzymes, known as synergism, is one of the most important phenomenon observed in cellulose degradation [1,18,20,50]. Synergistic action among different enzymes (such as endo-exo synergism, exo-exo synergism, exo-β-glucosidase synergism) have been studied; of these end-exo synergism is a highly effective synergism that has been reported in many studies [18,20,48,51].

**Figure 9:**
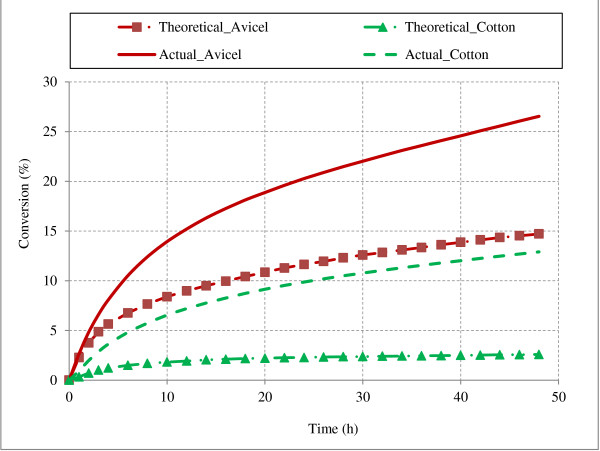
**Synergism between EG I and CBH I during hydrolysis of Avicel and cotton cellulose.** The figure illustrates the synergistic action of endoglucanases and cellobiohydrolase enzymes on Avicel and cotton cellulose. Solid lines are results from combined action of enzymes and lines with points (theoretical) are sum of conversions from action of individual enzymes. Hydrolysis conditions: 100 g/L, EG I 10 mg/g glucans, CBHI 10 mg/g glucans (total 20 mg enzymes when act in mixture 1:1).

Synergism between EG I and CBH I for Avicel and cotton cellulose was observed in model simulations (Figure 9). Simulations were performed for 48 hours assuming 100 g/L substrate concentration at enzyme loadings of 10 mg/g glucans (individually and total of 20 mg in mixture, with EG to CBH I ratio of 1:1). Comparison between theoretical conversion (sum of conversions from action of individual enzymes) and actual conversion (cellulose conversion from action of enzyme mixture) are presented in Figure 9.

One measure of synergism is “Degree of Synergism (DS)”, which is defined as follows (Equation 2)

(1) $$\mathrm{Degree}\;\mathrm{of}\;\mathrm{Synergism}\;=\;\frac{\Delta C_{\mathrm{mixed}}}{\sum_{i=1}^{n}\Delta C_{i}}$$

Where, ∆C_mixed_ is cellulose conversion obtained from mixture of ‘n’ enzymes; ∆C_i_ is cellulose conversion obtained from individual action of ‘i^th^’ enzyme.

For additional confirmation of synergistic action, two additional substrates filter paper and bacterial cellulose (substrate properties in Table 2) were simulated under similar conditions (100 g/L cellulose, 10 mg/g individual enzyme). Degrees of synergism from combined action of EGI and CBH I were calculated as 1.8, 1.9, 4.14 and 4.99 for Avicel, filter paper, bacterial cellulose and cotton respectively. The values of DS obtained from model simulations are consistent with the reported values in literature [20,33,37,48,51]. The inverse relationship between DS and substrate DP was expected and has been reported in literature [18,20,52]. Zhang et al. [20] compiled DS values from various studies (Table V of [20]) and reported DS values of 1.3 to 2.2 for Avicel and 4.1 to 10 for cotton and bacterial cellulose from synergism of *T.reesei* enzymes. When CBH I acts alone, its accessibility to chain ends is very limited and cellulose conversion is very less. During simultaneous action of EG and CBH I and/or CBH II, EG action results in creation of additional chain ends for action of CBH I and/or CBH II, which results in more effective hydrolysis (high synergism).

**Figure 10:**
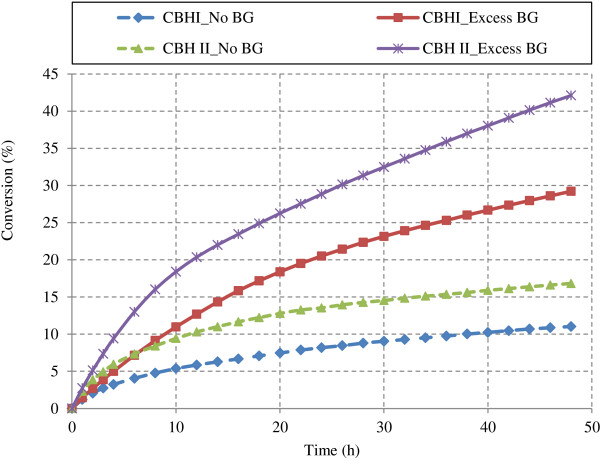
Effect of β-glucosidase addition on cellulose hydrolysis by CBH I and CBH II (Avicel, 100 g/L; CBH I, 10 mg/g glucans; CBH II, 10 mg/g glucans).

Another important synergism is between CBH I and/or CBH II and exo-β-glucosidase enzymes [20]. Synergism during Avicel hydrolysis by CBH I and CBH II in presence of β-glucosidase is reported in Figure 10. Primary product of CBH action on cellulose is cellobiose, a strong inhibitor to CBH activity [18-20,53,54]. Cellobiose build up is prevented by the action of β-glucosidase which further hydrolyzes cellobiose to glucose and results in CBH and β-glucosidase synergism. It can be observed from Figure 10 that model simulations capture this synergism successfully both for CBH I and CBH II enzymes. Cellulose conversions during Avicel hydrolysis (48 h) were observed 164.8 and 150.3% higher for CBH I (10 mg/g glucans) and CBH II (10 mg/g glucans) respectively in presence of excess β-glucosidase (200 IU/g glucans) than those in absence of β-glucosidase. Other than cellulose conversion, hydrolysis rate of Avicel by CBH I and CBH II with excess of β-glucosidase was markedly higher than that of CBH enzymes acting alone (data not reported).

### Cellulose hydrolysis by enzyme mixture

**Figure 11:**
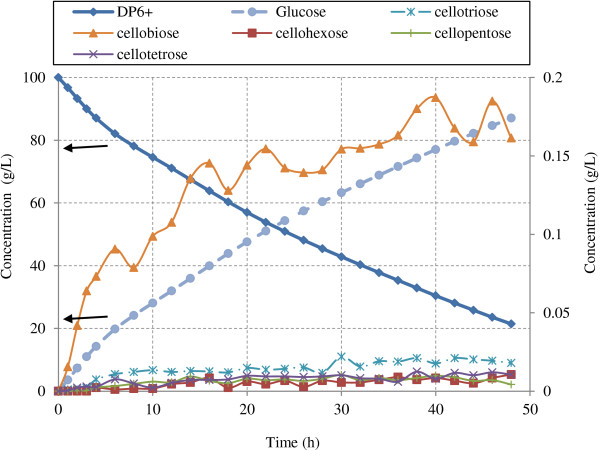
**Sugar concentrations during hydrolysis of Avicel (100 g/L) by protein mixture produced by *T.reesei*.** The figure illustrates the sugar production profile during Avicel hydrolysis by mixture of CBH I (60%), CBH II (20%), EGI (12%) at 20 mg protein/g glucans loading in excess of β-glucosidase (100 IU/g glucans). DP6+ and glucose are on primary axis and all other sugars are shown with respect to secondary y-axis.

**Figure 12:**
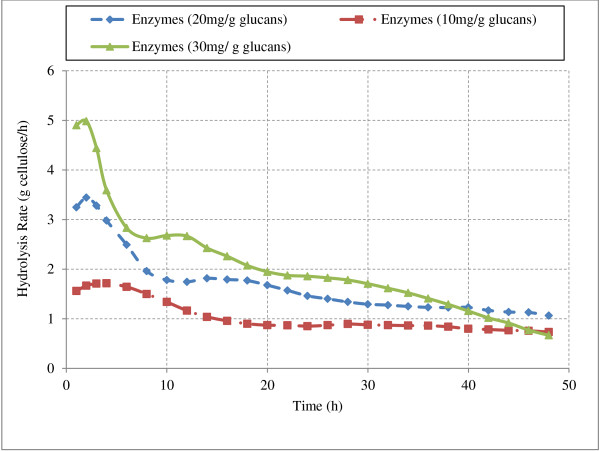
**Effect of enzyme loading on hydrolysis rate.** This figure shows the hydrolysis rate of Avicel at different loadings of enzyme mixture (12% EG I, 60% CBH I, 20% CBH II in presence of excess BG). Data used in the figure is provided in the Table A7.1 of Additional file 1.

Hydrolysis of Avicel was simulated using enzyme ratios similar to natural cellulase system produced by *T.reesei.* (12% EG I, 60% CBH I and 20% CBH II) [20,33,37]. Simulations were performed at various enzyme loadings (total protein of 10, 20, and 30 mg protein/g glucans) in excess of β-glucosidase to avoid cellobiose inhibition. Concentrations of various soluble sugars (DP 1–6) and hydrolysis rates during Avicel hydrolysis are presented in Figures 11 and 12 respectively.

Glucose was the main product of hydrolysis in presence of excess β-glucosidase which converts cellobiose to glucose (Figure 11). Although concentrations of all oligomers remained low throughout the hydrolysis, cellobiose concentration was highest among all oligomers. This trend was expected since cellobiose is the main product produced by the action of CBH I and CBH II while other oligomers are mainly produced by endoglucanases only. Similar results were obtained in terms of relative amount of oligomers from Avicel hydrolysis by same enzyme mixture in the absence of β-glucosidase. Cellobiose concentration was highest followed by cellotriose, which is also assumed to be not hydrolysable by either EG, CBH I or II. However cellulose conversion after 48 h of hydrolysis in absence of β-glucosidase was 33.4%, which was 57.5% less than that in presence of β-glucosidase (78.6%). This behavior was expected because as described earlier cellobiose produced by the action of CBH I and CBH II inhibits the activity of these enzymes and reduces overall cellulose conversion. Therefore for effective cellulose hydrolysis β-glucosidase enzyme is added to the cellulase preparation *T. reesei.* as the enzyme mixture produced by it has low levels of β-glucosidase [55]. Hydrolysis simulations were also performed under same hydrolysis conditions (*T. reesei*. enzyme mixture in excess of β-glucosidase) for cotton cellulose and the results of oligomer formation was found similar as in the case of Avicel hydrolysis. However cellulose conversion at end of 48 h hydrolysis was lower by 30.8% compared to Avicel. This observation can be attributed to the high crystallinity and higher degree of polymerization of the cotton cellulose compared to Avicel.

As expected, hydrolysis rates were found to be increasing with increase in enzyme loadings (Figure 12). For enzyme loading at 10 mg/g glucans hydrolysis rate remained constant during initial period followed by a substantial decrease and nearly constant rates thereafter. This behavior can be attributed to rapid decrease in the number of available surface glucose molecules with time. Number of accessible sites for endoglucanases decreases during hydrolysis. Initial constant rate phase was not observed at high enzyme loading (30 mg/g glucans) as all the accessible surface bonds were hydrolyzed at a very fast rate.

## Conclusions

Cellulose was modeled as group of microfibrils made of bundles of elementary fibrils. Model incorporated all important factors of cellulose hydrolysis such as structural features, morphological changes in cellulose during hydrolysis and effect of morphological changes on the hydrolysis. Model was accurate in predicting the cellulose hydrolysis profiles from experimental studies as well as followed other trends reported in literature studies. The major advantage of this model is that any change in substrate property, enzyme characteristics can be incorporated into model after performing independent characterization experiments. Other than predicting hydrolysis profiles, the model can be used to gain insight in the dynamic formation and breakdown of cello-oligomers during hydrolysis.

## Methods

### Model development

A SMM model that considers structural features of cellulose and complex enzyme-substrate interactions was developed. The SMM of cellulose hydrolysis process was systematically organized into three steps, consisting 1) *In-silico* construction of a representative cellulose polymer model, 2) characterization of the celluolytic enzymes in the mixture and 3) modeling the enzymatic hydrolysis.

#### Step 1: Cellulose polymer model

**Figure 13:**
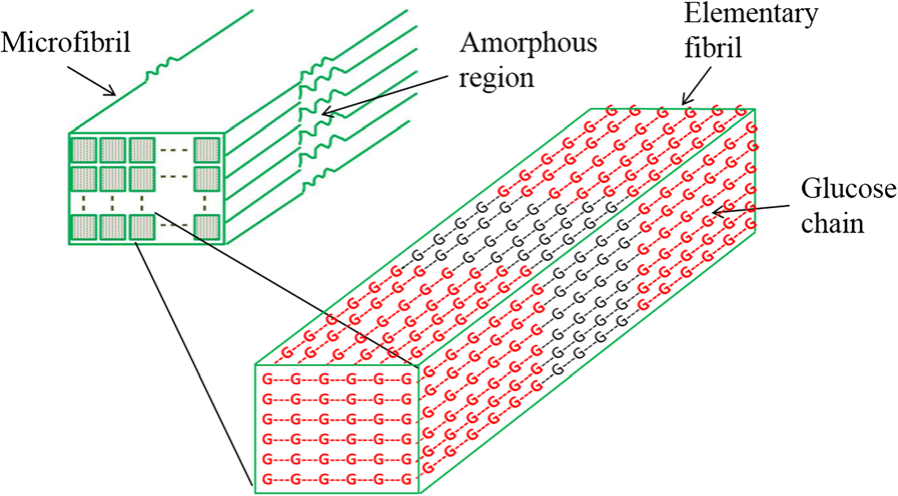
**Structure of microfibril and elementary fibril simulated in model.** Cellulose is group of microfibrils and each microfibril is group of elementary fibrils. Glucose molecules in red color are in crystalline region and glucose molecules in black color are in amorphous region.

Cellulose is found as several polymorphs [56-58] of which cellulose I_β_ is the most abundant form in higher plants, while I_α_ is the most common form in bacteria and several algae. In the cellulose I_β_ all glucose chains are assumed to be parallel (i.e. all reducing ends are on one side) while the I_α_ consists of glucose chains in anti-parallel arrangement. Basic building block of cellulose in all natural sources of cellulose is about 3.5 nm diameter elementary fibril made of 36 glucose chains. These EFs are organized into microfibrils (2–20 nm diameter) which in turn form macrofibrils [59]. Inter-fibril space is filled with different proportions of hemicellulose and lignin depending on the biological origin of the biomass [60-62]. The degree of polymerization of glucose chains and crystallinity of cellulose are dependent on biological origin and form of cellulose (pure or modified). In this model, cellulose I_β_ was modeled as group of microfibrils. Each microfibril contains several elementary fibrils [1,54,59] and DP of all EFs in one MF was assumed same. MF was represented as two dimensional matrix (bundle) containing several EFs. Each elementary fibril in turn was represented as a 3-D matrix with 36 glucose chains and each chain having glucose molecules equal to DP. Figure 13 illustrates the structure of a microfibril and elementary fibrils simulated in the model. Cellulose is semi-crystalline in nature and contains both amorphous (less ordered) and crystalline (ordered) regions [3,54,56,59]. Degree of crystallinity may vary depending upon the origin of cellulose and can vary between 50 and 90% [63]. Explanation about arrangement of crystalline and amorphous regions in the cellulose molecules is not definitively known. However, most commonly assumed cellulose structures depict glucose chains passing through several crystalline and amorphous regions [19]. To capture this characteristic in this model, cellulose was modeled in way that each glucose chain passes through multiple crystalline regions (200 glucose molecules long regions) separated by amorphous regions.

Crystallinity was characterized using a term crystallinity index (CrI) which was defined as:

(2) $$\mathrm{CrI}=\frac{\mathrm{Glu}\cos e\;\mathrm{molecules}\;\mathrm{in}\;\mathrm{crystalline}\;\mathrm{region}}{\mathrm{Total}\;\mathrm{glu}\cos e\;\mathrm{molecules}\;\mathrm{in}\;\mathrm{insoluble}\;\mathrm{cellulose}}$$

**Figure 14:**
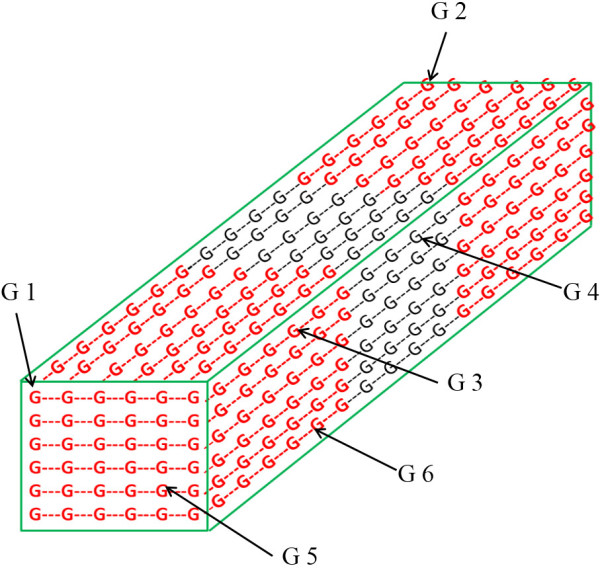
**Structure of elementary fibril simulated in model.** The figure illustrates the arrangement of glucose molecules in an elementary fibril. Glucose molecules in red color represents crystalline region and glucose molecules in black color are in amorphous region.

Each glucose molecule in a microfibril has a unique serial number as its identity. Several other parameters that describe structural properties of that bond were allotted to all glucose molecules (Figure 14 and Table 3 illustrate the approach for few parameters). Detailed description of all parameters is provided in supplementary material (Section A1 of Additional file 1).

**Table 3:** **Parameters of glucose molecules in an elementary fibril (corresponds to Figure**14**)**

**Properties →**	**Serial Number**	**Reducing/Non-reducing**^**a**^	**EF Surface**^**b**^	**Crystalline**^**c**^	**Soluble**^**d**^	**Distance_NR**^**e**^
G1	0	1	1	1	-1	16
G2	16	-1	1	1	-1	0
G3	88	0	1	1	-1	12
G4	94	0	1	0	-1	7
G5	476	1	0	1	-1	16
G6	600	0	1	1	-1	11

^a^ Indicates whether bond is reducing end (1), non-reducing end (-1) or inside the chain (0).

^b^ Indicates whether bond is at elementary fibril surface or not (Yes, 1 or No, 0).

^c^ Indicates whether bond is in crystalline (1) or amorphous region (0).

^d^ Indicates whether bond is in soluble (1), partially soluble (0) or insoluble chain (-1).

^e^ Indicates the distance of bond from non-reducing end.

These parameters were used to determine accessibility of enzymes depending upon their action pattern and directly affect the hydrolysis process. For example it is important to consider bond location (amorphous or crystalline region) since activity of endoglucanases enzymes is relatively low in crystalline region [18,20]. Similarly not all endoglucanases produce glucose and the parameter “Distance_NR” helps modeling this case. EF surfaces are less accessible to enzymes compared to MF surfaces. The properties “EF Surface” (Table 3) and “MF Surface” help in identifying the bond location and simulating enzymatic action appropriately. While assigning these parameters, it was ensured that cellulose model captures all major structural properties of actual cellulose. For instance, the crystalline and amorphous regions were considered to occur as bands in elementary fibril. While the length of the crystalline region was set to 200 glucose molecule, the location was randomly chosen along the length of the elementary fibril such that the amorphous regions do not occur at the ends of the elementary fibril (Figure 1).

#### Step 2: Characterization of cellulolytic enzyme system

Cellulase enzymes have different mode of actions and cannot be strictly placed into broad groups such as endoglucanases and exoglucanases [20]. In this model, enzymes were classified into eight classes depending upon their structure and mode of action (Table 4).

**Table 4:** Mode of action for enzymatic hydrolysis of cellulose by enzymes from various classes

**Enzyme class**	**Mode of action**
Endo-cellulase (EG, Non-processive with CBM)	• Carbohydrate binding module (CBM) binds to surface chains randomly and breaks bonds at catalytic domain (CD)
Endo-cellulase (EG, Non-processive without CBM)	• Breaks bonds on surface chains in a random pattern
Endo-cellulase (EG, Processive with CBM)	• CBM binds to surface chains randomly and break bond at CD
	• Enzyme moves along the chain (towards non-reducing end or reducing end) and cuts every alternate bond releasing cellobiose until a minimum chain length is achieved
Exo-cellulase (CBH I, Processive)	• CBM attaches from reducing end only on surface chains and pulls the chain towards CD
	• Chain passes through CD (tunnel like shape) and every alternate bond is broken to produce cellobiose
	• Enzyme moves along the chain (towards non-reducing end) and cut every alternate bond until a minimum chain length is reached
Exo-cellulase (CBH I, Non-Processive)	• Attack from reducing end on surface chains and cuts every alternate bond to produce cellobiose
Exo-cellulase (CBH II, Processive)	• CBM attaches from non-reducing end on surface chains and pulls the chain towards CD
	• Chain passes through the tunnel shaped CD and every alternate bond is broken to produce cellobiose
	• Enzyme moves along the chain (towards reducing end) and cuts every alternate bond until a minimum chain length is reached
Exo-cellulase (CBH II, Non-Processive)	• Attack from non-reducing end on surface chains and breaks alternate bonds to produce cellobiose
β-glucosidase	• Acts on cellobiose and soluble oligomers (DP ≤ 6) and produce glucose by breaking bond

CBM - Carbohydrate binding module CD - Catalytic domain.

There can be multiple enzymes in each class identified from different organisms; however they will differ only in their hydrolytic efficiency (activity). Therefore modeling the action of these eight classes of enzymes will sufficiently capture the dynamics individual cellulases. The important aspect of this classification is that any new enzyme can be used in model after characterizing its action pattern and placing into one/multiple groups with its specific activity /activities, without making any change to the current model.

#### Step 3: Modeling the action of enzymes

Factors affecting enzyme action can be classified as intrinsic and extrinsic factors. Intrinsic factors do not depend on substrate characteristics while extrinsic factors depend solely on substrate characteristics. Extrinsic factors (crystallinity, accessibility and average DP) are accounted in the cellulose model described in Step 1. Intrinsic characteristics such as activity and stability of the enzyme are dependent on the reactor pH and temperature. The enzyme loading (amount of enzyme/g substrate) and activity information was transformed into number of bonds hydrolyzed per unit time for each enzyme.

Maximum number bonds (N_hi_max_) that can be broken by an enzyme per minute was calculated using equation 3.

(3) $$N_{\mathrm{hi}\_\max}=E_{i}*U_{i}*6.023*10^{17}*\frac{G_{\mathrm{Sim}}}{6.023*10^{23}}*162*S_{i}$$

Where, 'E_i_' is amount of 'i^th^' enzyme used per unit of cellulose during hydrolysis, mg/g cellulose. 'U_i_' is activity of 'i^th^' enzyme, IU/mg enzyme. One international unit (IU) of enzyme can liberate one micromole (6.023 * 10^17^ molecules) of product (i.e. one micromole of bonds are broken) per minute under standard conditions [64]. 'G_sim_' is the number of glucose molecules simulated in the model. “162” is the average molecular weight of anhydrous glucose molecule in a cellulose molecule (joined by β-1-4 linkages in long chains with DP from 100 to 20000). 'S_i_' is stability of 'i^th^' enzyme under current hydrolysis conditions (temperature and pH) that will affect the activity. Value of “S_i_” can be calculated for any enzyme using empirical equations developed based on experiments to incorporate the effect of temperature and pH on enzyme activity. For example, Arrhenius rate relationships are commonly used to consider the effect of temperature [22]. Value of 'S_i_' is a real number between 0 and 1. In the current model, value of “S_i_” was used as 1, indicating that the enzyme was considered to be operating at its optimum pH and temperature.

Assuming enzymes have equal access to all microfibrils, number of maximum bonds that can be broken for each microfibril (N_hij_) were calculated as:

(4) $$N_{\mathrm{hij}}=N_{\mathrm{hi}\_\max}*f_{j}$$

Where, f_j_ is fraction of hydrolysable bonds for 'j^th^' microfibril and is dependent upon mode of action of enzymes.

For EG enzymes (Non-processive endoglucanase with CBM, Non-processive endoglucanase without CBM, Processive endoglucanase with CBM), f_j_ was defined as:

(5) $$f_{j}=\frac{N_{\mathrm{sj}}}{\sum_{1}^{n}N_{\mathrm{sj}}}$$

Where, N_sj_ is number of glucose molecules on the surface of elementary fibrils in j^th^ microfibril and 'n' is number of microfibrils simulated.

For CBH I enzymes (Processive CBH I with CBM, Non-processive CBH I without CBM), f_j_ was defined as:

(6) $$f_{j}=\frac{N_{\mathrm{Rj}}}{\sum_{1}^{n}N_{\mathrm{Rj}}}$$

Where, N_Rj_ is number of reducing ends in j^th^ microfibril.

For CBH II enzymes (Processive CBH II with CBM, Non-processive CBH II without CBM), f_j_ was defined as:

(7) $$f_{j}=\frac{N_{\mathrm{NRj}}}{\sum_{1}^{n}N_{\mathrm{NRj}}}$$

Where, N_NRj_ is number of non-reducing end in j^th^ microfibril.

For β-glucosidase (BG) enzymes, f_j_ was defined as:

(8) $$f_{j}=\frac{N_{\mathrm{SLj}}}{\sum_{1}^{n}N_{\mathrm{SLj}}}$$

Where, N_SLj_ is number of soluble oligomers produced from j^th^ microfibril.

#### Enzymatic Hydrolysis Simulation

Action of various cellulase enzymes on cellulose structure was modeled using Monte Carlo simulation technique [38-40,42]. Each minute, a sequence of *N*_*hij*_ potential hydrolysis bond locations were randomly selected (with uniform probability distribution) from the list of bond locations appropriate for the type of enzyme under consideration. For example, in case of EG and CBH, random bond locations were generated from list of bonds present on the EF surface, while for BG, the bond locations were selected from the list of soluble molecules only. In each iteration location of bond inside a microfibril corresponding to randomly chosen bond location (glucose molecule) was determined and its properties (by associated parameters) for specific enzyme action were examined to determine the probability of hydrolysis. Enzymatic hydrolysis is controlled by both enzyme action and structural physical properties of cellulose; conditions for enzyme actions were simulated accordingly. All the required substrate-enzyme interaction characteristics were incorporated into the model. For example, CBH I will bind only if there is a reducing end (non-reducing end for CBH II) at the chosen target location for binding. Similarly, probability of binding on molecules located on a microfibril surface was set higher than that of molecules on interior elementary fibril surface to account for differences in accessibility of bonds to enzymes. It was also ensured that sufficient unobstructed chain length (measured in terms of number of glucose molecules corresponding to the size of the enzyme CBM, linker and CD) is available on the surface of the substrate for CBM binding. A summary of some important conditions for enzymes from endo, exo and β-glucosidase have been presented in Table 5. Some details on enzyme binding and action simulated in the model have been provided in the supplementary material (Section A2 of Additional file 1). During this one minute of hydrolysis, action of all enzymes was randomized (i.e. enzyme from different classes do not attack in a particular sequence) to enable realistic simulation of simultaneous action of enzymes.

**Table 5:** Important conditions simulated in the model for enzymatic hydrolysis of cellulose by enzymes from endo, exo and β-glucosidase classes

**Enzyme class**	**Conditions/assumptions**
Endo-cellulase (EG, Non-processive action pattern by an enzyme that contains a CBM)	• Lower probability of breaking bond in crystalline region compared to those in amorphous regions
	• Probability of binding to the glucose chain and hydrolysis of bonds located on EF surface but not on MF surface is 25% lower compared to the glucose chains on the both EF and MF surfaces
	• A minimum number of glucose molecules, depending upon size of enzyme, are required to be unblocked (not blocked by other enzymes) and on the EF surface to enable binding (please see section A3 of Additional file 1)
	• Enzymes do not act on oligomers of chain length less than five. The enzymes can hydrolyze bonds to produce glucose with a defined 90% probability. Inhibition occurs due to cellobiose and glucose [1,54]
Exo-cellulase (CBH I, Processive action pattern by an enzyme that may/may not have a CBM.)	• Probability of binding to the glucose chain and hydrolysis of bonds located on EF surface but not on MF surface is 25% lower compared to the glucose chains on the both EF and MF surfaces
	• These enzymes have higher probability of hydrolysis with shorter glucose chains compared to longer chains [20]. A preferred chain length of 300 glucose molecules was assumed
	• Enzyme may get desorbed from the glucose chains at any time. Probability of desorption in amorphous region (5.0%) was set higher than that in crystalline region (2.5%)
	• Enzymes have lower probability of bond hydrolysis in the case of soluble oligomers and do not act on oligomers with DP<5
	• Inhibition occurs due to cellobiose and glucose, however cellobiose is a stronger inhibitor [1,19,50,54]
β-glucosidase	• Acts only on soluble chains (DP<6)
	• Probability of action on cellobiose is higher than other soluble oligosaccharides
	• Inhibited by glucose

In addition to the mode of specific action pattern of enzymes (Endo/Exo-enzymes; processive or non-processive) probability of hydrolysis was set to be dependent on enzyme characteristics such as the bond location (crystalline or amorphous region) and soluble oligomer chain length. For example, during the action by EG enzymes, the probability of β-1,4 bond hydrolysis located in crystalline and amorphous regions are different. Similarly, the probability of bond hydrolysis in soluble chains with lower DP was set lower compared to the probability of hydrolysis for chains with higher DP. These probabilities were compared with a number randomly selected from a uniform distribution. The bond at a particular location was hydrolyzed only if the random number (generated from a uniform pseudo-random number distribution 0.0-1.0) was greater than the probability of hydrolysis set using the constraints described above. For example a bond located in the crystalline region could be broken (hydrolysis event occurred) only if the probability of the hydrolysis (as indicated by the pseudo-random number from the uniform distribution 0.0-1.0) was higher than the set value for probability of crystalline bond hydrolysis by EG enzymes. Similarly EG enzyme will hydrolyze a bond next to the reducing end or the bond prior to the non-reducing end (hydrolysis of this bond will produce glucose) only if its associated probability is higher than pseudo-random number.

**Figure 15:**
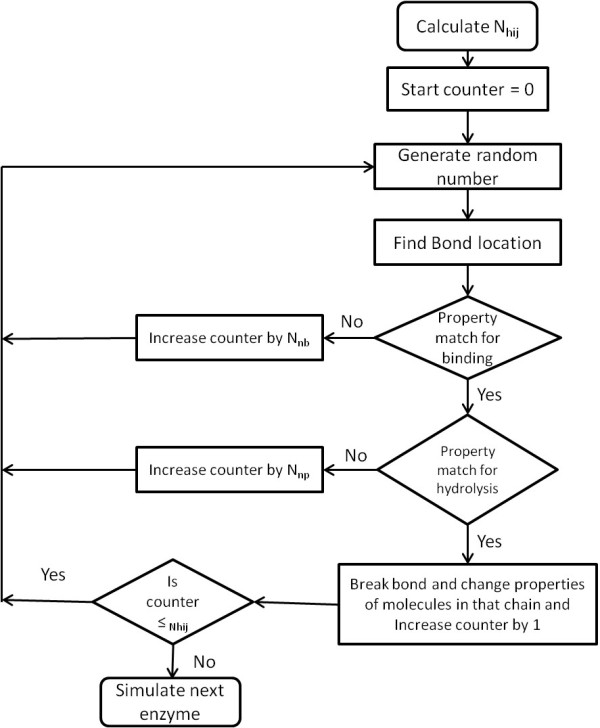
**Schematic for hydrolysis simulations in model.** The figure illustrates the steps followed during hydrolysis simulations of individual enzyme acting on cellulose. “N_hij_” is maximum number of bonds an enzyme can break per minute in one microfibril (‘jth’ MF); ‘N_nb_’ is counter increment when no binding occurs; ‘N_np_’ is counter increment in case of non-productive binding when binding occurs without bond hydrolysis.

A schematic of steps followed during hydrolysis a simulation in the model is shown in Figure 15. A counter was used during one minute of hydrolysis to limit the number of iterations. As discussed earlier, a random number was generated from a group of molecules and bond properties were checked for hydrolysis possibility. If all conditions for hydrolysis were met for that bond, it was converted to broken bond (hydrolysis event occurred) and counter was changed (values are given in section A3 of Additional file 1). In case of non-processive enzymes, a random number is generated again to find next bond location, whereas for processive enzymes, enzyme hydrolyzes the next bond unless it is desorbed from the chain or counter value is equal to N_hij._

Inhibition effect of sugars produced was captured in the model using the same approach. Activities of both CBH and endoglucanases are inhibited by cellobiose and glucose. Inhibition properties for cellobiose (N_inhib _G2_) and glucose (N_inhib _G_) were assigned to each class of enzyme. During hydrolysis simulations, whenever bond location determined using random number generated was corresponding to glucose or cellobiose, counter was incremented by corresponding “N_inhib_” for that enzyme. As the hydrolysis progresses, probability of encountering glucose or cellobiose increases, which results in less number of bonds hydrolysis (as counter is incremented by “N_inhib_”) and inhibition effect is captured. Extent of inhibition can be controlled by adjusting these properties. For example, cellobiose is strong inhibitor compared to glucose for CBH [1,19,27,50,54]; therefore value of “N_inhib _G2_” was set higher compared to “N_inhib _G_” for cellobiose in the model.

Properties of all glucose molecules (mentioned in Table 3 and section A1 of Additional file 1) in the chain, where bond is broken are reassigned after productive attack (hydrolysis event occurs). Initially, only the chains on the elementary fibril surface are accessible to enzyme attack. During hydrolysis, as the bonds are broken, glucose chains having DP less than seven become soluble (i.e. soluble oligomers are removed from the surface of the cellulose) and part of chain just beneath the soluble chain is exposed and becomes accessible to enzymes.

Sugar concentrations were calculated at various time intervals to predict the hydrolysis profile during model simulations. Concentrations of glucose, cellobiose and insoluble sugars were calculated using following equations (equations 9–11).

(9) $$C_{\mathrm{Glu}}=\frac{N_{\mathrm{Glu}}*G_{\mathrm{actual}}}{G_{\mathrm{sim}}}*\frac{180}{6.023*10^{23}}*\frac{1000}{V_{\mathrm{actual}}}$$

(10) $$C_{G2}=\frac{N_{G2}*G_{\mathrm{actual}}}{G_{\mathrm{sim}}}*\frac{342}{6.023*10^{23}}*\frac{1000}{V_{\mathrm{actual}}}$$

(11) $$C_{G6+}=\frac{N_{G6+}*G_{\mathrm{actual}}}{G_{\mathrm{sim}}}*\frac{162}{6.023*10^{23}}*\frac{1000}{V_{\mathrm{actual}}}$$

Where, 'C_Glu_', 'C_G2_', 'C_G6+_' are concentrations of glucose, cellobiose, and high DP molecules in gram/L respectively. 'G_actual_' is number of glucose molecules in actual sample (experimental conditions) (equation 12). 'G_sim_' is number of glucose molecules simulated in the model. 'V_actual'_ is volume of solution in mL.

(12) $$G_{\mathrm{actual}}=\frac{W_{\mathrm{sample}}*S*C*6.023*10^{23}}{162}$$

Where, 'W_sample_' is weight of total solution during hydrolysis in grams. 'S' is fraction of solids in the solution (biomass or solid loading), dimensionless. 'C' is cellulose fraction of the solid, dimensionless. Explanation about these calculations and of other oligomers is provided in detail in the supplementary material (Section A4 of Additional file 1).

The hydrolysis rates were estimated by calculating the changes in cellulose concentration (DP 6+) over a specific time interval and was expressed as g cellulose/h.

Chain distribution provides the number of chains with different chain lengths in total cellulose, which can help in understanding of hydrolysis process. Therefore chain distribution profile was generated at specific time intervals. Additionally, crystallinity index and percentage of soluble molecules were also calculated at specific time intervals to monitor the changes in these properties.

### Implementation of model

The computer algorithms for implementation of above described hydrolysis model were written in C++ language. Random number generators were used in simulation of cellulose structure and hydrolysis process [65].

Cellulose structure was simulated using user defined range of structural parameters: DP, CrI, number of EFs in one microfibril. This approach allows simulation of any cellulose substrate such as Avicel, filter paper (with low DP and crystallinity), bacterial cellulose, cotton fibers (long DP and highly crystalline). Hydrolysis simulation was initialized based on the weight of solution (scale of hydrolysis), solid loading, cellulose content, total enzyme loading (mg protein/g cellulose), ratio of enzymes present (out of eight classes), temperature, pH and simulation time (hydrolysis duration). Enzyme activities can be obtained from manufacturer’s data or measured on different substrates (cellulose model substrates) using standard laboratory methods [64]. Maximum value of enzyme activity is used in the model. Data provided by user on enzyme ratio was input to model through data files. The output from model included glucose concentrations, oligosaccharide concentrations (DP 2–6), chain distribution profile, crystallinity index profile, solubility profile and data sheets for each microfibril (illustrating major properties associated with glucose molecules) at various times during hydrolysis.

The approach of using various probabilities (such as endo enzyme acting on crystalline region, cutting the bond just before non-reducing end to produce glucose) provides flexibility to simulate various enzymes and to incorporate new enzymes in the model. For example, in general EG acts preferentially in amorphous region, however in future an enzyme may be discovered/developed with high activity in crystalline region. That enzyme can be easily incorporated into current model by changing the probability for bond cleavage in the crystalline regions. Similarly other probabilities can be altered depending upon enzyme characteristics.

### Model validation and simulations

#### Simulation data

Cellulose structure was simulated for various model cellulose substrates such as Avicel, bacterial cellulose (BC), cotton. These substrates differ in cellulose structure in terms of DP and degree of crystallinity. For instance, Avicel is low DP cellulose (about 300), whereas cotton has very high DP value (about 2000) [20]. Values of properties used to simulate these model cellulosic substrates are listed in Table 2[20,33]. Multiple microfibrils were simulated for each model substrate using these properties and assuming 4–6 EF in each row and column of microfibril. A minimum of 1,000,000 total glucose molecules were simulated to represent any cellulose substrate during hydrolysis simulations. These model substrates can vary somewhat in terms of DP, crystallinity index depending upon method of preparation (e.g. Avicel will not always have DP of exact 300), therefore using a range of properties and simulating multiple microfibrils captures this variability.

All model simulations were performed for four main classes of cellulase enzymes: Non-processive endoglucanases with CBM, processive CBH I with CBM and processive CBH II with CBM and β-glucosidase. These enzymes are similar to enzyme system of *Trichoderma reesei* (earlier known as *T. viride*), comprising endoglucanases EGI, two exoglucanases CBHI and CBHII and β-glucosidase. Cellulase system of *T. reesei*, a soft-rot fungi, has been extensively studied [1,3,18,20]. Specific activities of enzymes from *T. reesei* were assumed as 0.4, 0.8, and 1.6 IU/mg of EG I, CBH I and CBH II respectively [33]. Zhang and Lynd [33] calculated these specific activities values from various literature studies [66-68].

#### Model validation

Data from model simulations were compared with two sets of experimental results from literature to validate the model.

#### Validation data set 1: Hydrolysis of Avicel

Experimental details for the experimental data are detailed in Bezerra et al. [45-47]. Briefly, Avicel was hydrolyzed using CBHI (Cel7A) purified from Celluclast 1,5 L (a commercial cellulase preparation from *T. reesei*, provided by Novo Nordisk A/S, Denmark), at various enzyme: substrate loadings at 40 °C in 50 mM citrate buffer (pH 4.8) for 47 h.

#### Validation data set 2: Hydrolysis of Avicel

For model validation, cellulose conversion results were extracted from Figure 3 of Medve et al. [48]. Briefly, Avicel (M2331from Merck, Darmstadt, Germany) was hydrolyzed using CBH I and EG II (8 mg EGII/g Avicel and 8 mg CBHI/g Avicel), purified from a commercial cellulase, Celluclast from *T. reesei* (Novo Nordisk, Bagsvaerd, Denmark). For model simulations, activity of EG II enzymes used in experiments were assumed to be same as EG I enzyme (0.4 IU/mg protein).

## Abbreviations

∆Cmixed: Cellulose conversion by action of mixture of ‘n’ enzymes; ∆Ci: Cellulose conversion by individual action of ‘i^th^’ enzyme; BC: Bacterial cellulose; BG: β-glucosidase; C: Cellulose fraction of the solid; CBH: Cellobiohydrolases; CBM: Carbohydrate binding module; CBP: Consolidated bioprocessing; CD: Catalytic domain; CGlu: Concentration of glucose (g/L); CG2: Concentration of cellobiose (g/L); CG6+: Concentration of high DP molecules (g/L); CrI: Crystallinity index; DP: Degree of polymerization; DS: Degree of synergism; EF: Elementary fibril; EG: Endoglucanses; Ei: Amount of 'i^th^' enzyme used per unit of cellulose during hydrolysis (mg/g cellulose); fj: Fraction of hydrolysable bonds for 'j^th^' microfibril; Gactual: Number of glucose molecules in actual sample; Gsim: Number of glucose molecules simulated in the model; IU: International unit of enzyme activity; MF: Microfibrils; n: Number of microfibrils simulated; Nhij: Number of maximum bonds broken by i^th^ enzyme on j^th^ microfibril; Nhi_max: Maximum number bonds broken by an enzyme per minute; Ninhib: Increment in counter due to inhibition; Ninhib _G2: Increment in counter due to cellobiose encounter/inhibition; Ninhib _G: Increment in counter due to glucose encounter/inhibition; Nnb: Increment in counter when no binding occurs; Nnp: Increment in counter in case of non-productive binding; NNRj: Number of non-reducing end in j^th^ microfibril; NRj: Number of reducing ends in j^th^ microfibril; Nsj: _N_umber of glucose molecules on the surface of elementary fibrils in j^th^ microfibril; NSLj: Number of soluble oligomers produced from j^th^ microfibril; ODE: Ordinary differential equations; R2: Square of the Pearson product–moment correlation coefficient; S: Fraction of solids in the solution; Si: Stability of 'i^th^' enzyme under current hydrolysis conditions; SMM: Stochastic molecular modeling; T. reesei: *Trichoderma reesei*; Ui: Activity of 'i^th^' enzyme (IU/mg enzyme); Vactual: Volume of solution (mL); Wsample: Weight of total solution during hydrolysis (g)

## Competing interests

The authors declare that they have no competing interests.

## Authors’ contributions

DK developed the model, carried out the process simulations and wrote the paper. GM conceived and designed the study, helped in the development of the model, and reviewed the results and manuscript. All authors read and approved the final manuscript.

## Authors’ details

^1^Graduate Student, Biological and Ecological Engineering, 116 Gilmore Hall, Oregon State University, Corvallis, OR-97331. ^2^Assistant Professor, Biological and Ecological Engineering, 116 Gilmore Hall, Oregon State University, Corvallis, OR-97331.

## Supplementary Material

Additional file 1 **This file includes seven sections (A1 – A7) as follow: Section A1:** Parameters associated with glucose molecules in the model. This section illustrates the properties associated with glucose molecules in the model. **Section A2:** Binding and action of endoglucanase and cellobiohydrolase enzymes. This section explains the action pattern of cellulase enzymes on cellulose. **Section A3:** Values of parameters used for EG I, CBH I and CBH II action. This section provides the values of parameters (such as increment on productive or non-productive binding) used in model simulations. **Section A4:** Calculations of concentrations of soluble and insoluble sugars. Equations used to calculate concentration of soluble and insoluble sugars during hydrolysis are presented in this section. **Section A5:** Cellobiose production during hydrolysis of Avicel by CBH I action. This section presents the data from model simulation from hydrolysis of Avicel at various enzyme:substrate ratios. Simulations were performed three times at each condition and standard deviations are provided in the table. **Section A6:** Endoglucanases action on substrates with different crystallinity. This section illustrates effect of crystallinity on the hydrolysis profile of cellulose by endoglucanases action. **Section A7:** Effect of enzyme loading on the hydrolysis rate of cellulose. This section presents the data from model simulation from hydrolysis of Avicel at various enzyme loadings (data used for Figure 15). Click here for file
