# Supplementary material for: Stochastic molecular model of enzymatic hydrolysis of cellulose for ethanol production
Source: Biotechnol Biofuels. 2013 May 2;6:63. doi: 10.1186/1754-6834-6-63 (PMC3664589; doi:10.1186/1754-6834-6-63)
Supplement: Additional file 1 — This file includes seven sections (A1 – A7) as follow: Section A1: Parameters associated with glucose molecules in the model. This section illustrates the properties associated with glucose molecules in the model. Section A2: Binding and action of endoglucanase and cellobiohydrolase enzymes. This section explains the action pattern of cellulase enzymes on cellulose. Section A3: Values of parameters used for EG I, CBH I and CBH II action. This section provides the values of parameters (such as increment on productive or non-productive binding) used in model simulations. Section A4: Calculations of concentrations of soluble and insoluble sugars. Equations used to calculate concentration of soluble and insoluble sugars during hydrolysis are presented in this section. Section A5: Cellobiose production during hydrolysis of Avicel by CBH I action. This section presents the data from model simulation from hydrolysis of Avicel at various enzyme:substrate ratios. Simulations were performed three times at each condition and standard deviations are provided in the table. Section A6: Endoglucanases action on substrates with different crystallinity. This section illustrates effect of crystallinity on the hydrolysis profile of cellulose by endoglucanases action. Section A7: Effect of enzyme loading on the hydrolysis rate of cellulose. This section presents the data from model simulation from hydrolysis of Avicel at various enzyme loadings (data used for Figure 15). [file 1754-6834-6-63-S1.pdf]

Appendix

Stochastic Molecular Model of Enzymatic Hydrolysis of Cellulose for Ethanol Production

Deepak Kumar[1], Ganti S. Murthy[1*]

[1]Biological and Ecological Engineering, Oregon State University, Corvallis, USA

*Corresponding Author

Ganti S. Murthy

Assistant Professor

Biological and Ecological Engineering

116 Gilmore Hall, Oregon State University, Corvallis, OR-97331.

Ph. - 541-737-6291

murthy@engr.orst.edu

This Appendix of supporting information consists of 19 pages that include seven sections (A1 – A7) as follow:

Section A1: Parameters associated with glucose molecules in the model. This section illustrates the properties associated with glucose molecules in the model.

Section A2: Binding and action of endoglucanase and cellobiohydrolase enzymes. This section explains the action pattern of cellulase enzymes on cellulose.

Section A3: Values of parameters used for EG I, CBH I and CBH II action. This section provides the values of parameters (such as increment on productive or non-productive binding) used in model simulations.

Section A4: Calculations of concentrations of soluble and insoluble sugars. Equations used to calculate concentration of soluble and insoluble sugars during hydrolysis are presented in this section.

Section A5: Cellobiose production during hydrolysis of Avicel by CBH I action. This section presents the data from model simulation from hydrolysis of Avicel at various enzyme:substrate ratios. Simulations were performed three times at each condition and standard deviations are provided in the table.

Section A6: Endoglucanases action on substrates with different crystallinity. This section illustrates effect of crystallinity on the hydrolysis profile of cellulose by endoglucanases action.

Section A7: Effect of enzyme loading on the hydrolysis rate of cellulose. This section presents the data from model simulation from hydrolysis of Avicel at various enzyme loadings (data used for figure 12).

Section A1: Parameters associated with glucose molecules in the model

Several parameters were assigned to each glucose molecule in the microfibril of cellulose that describe structural properties of that bond. These properties were used to determine accessibility of enzymes depending upon their action pattern and directly affect the hydrolysis process. Descriptions of all parameters associated with each glucose molecule are provided below.

- **Serial number**: Each glucose molecule in the microfibril has unique serial number as its identity. Numbering of molecules starts from first glucose molecule of first glucose chain of first elementary fibril (reducing end). Numbering was continued from chain to chain till all molecules in one elementary fibril were numbered and was continued to next elementary fibril.
- **Reducing/Non-reducing**: Bond is located at the reducing end (value of "1"), non-reducing end (value of "-1") or somewhere inside the chain (value of "0")
- **Hydrolyzable**: Bond is hydrolysable (value of "1") or has been already hydrolyzed (value of "0")
- **MF_surface**: Bond is on microfibril surface (Yes, 1 or No, 0)
- **EF_surface**: Bond is on elementary fibril surface (Yes, 1 or No, 0)
- **Crystalline**: Bond is in crystalline (value of "1") or amorphous region (value of "0")
- **Chainlength**: Parameter indicates the length of chain in which this glucose molecule exists
- **Soluble**: Indicates whether bond is in soluble (DP<=6, value of "1"), partially soluble (DP from 6 to 13, value of "0") or insoluble chain (DP>13, value of "-1")
- **Distance_NR**: Indicates the distance of bond from non-reducing end. When used in conjunction with the Chainlength property, this can be used to reallot the properties to glucose molecules in the chain where hydrolysis occurs (bond is broken)
- **Blocked**: Indicates whether bond is blocked (some enzyme already bound to the bonds in the neighborhood of this bond) or is free ("0" for free and different numbers for different class of enzymes)

Section A2: Binding and action of endoglucanase and cellobiohydrolase enzymes

Action of cellulase enzymes simulated in the model is illustrated in figure A2.1. Endoglucanase bind randomly along the surface glucose chains and hydrolyze one/few accessible bonds. Thus endoglucanases results in rapid decrease in the degree of polymerization (DP) of cellulose.

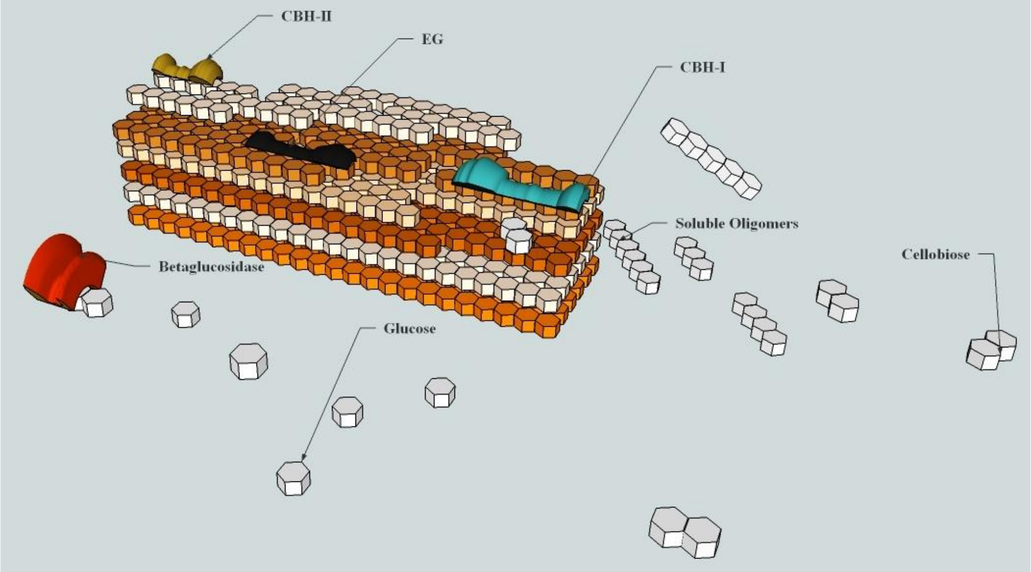

Figure A2.1 Action pattern of cellulose enzymes simulated in the model

An exoglucanase, a processive enzyme, attacks from the chain ends (CBH I from reducing end and CBH II from non-reducing end) and produces cellobiose as main product. Processive exoglucanases continue hydrolysis of other glycosidic bonds until they reach the end of the chain while non-processive enzymes are desorbed after hydrolysis of each bond [1]. β-glucosidases hydrolyze the cellobiose and short soluble oligomers to glucose and complete the hydrolysis process [2]. Most of the endoglucanases and cellobiohydrolase enzymes contain two independent domains or modules: carbohydrate binding module (CBM) and catalytic domain (CD). These domains are joined by peptide linker. The cellulase shape used in the model simulations

contained all three modules (Fig. A2.2) and was designed with some modifications from that of Levine et al [3].

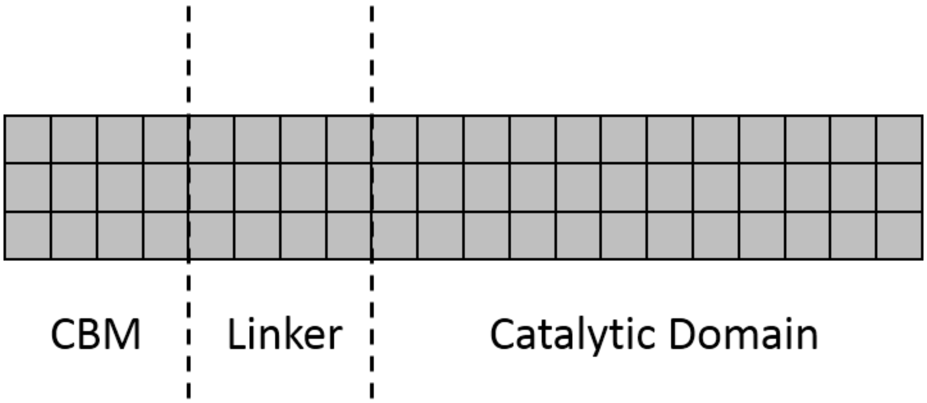

Figure A2.2 Shape of cellulase used in the model. Each block represents a glucose molecule (modified from Levine et al.)

CBM binds to molecules and helps in hydrolysis by bringing the high local concentration of enzymes close to surface and providing more time to the enzyme in close proximity of the substrate [4-7]. It has been also suggested that CBM affects cellulose structure by disrupting the hydrogen bond network, modifying spatial arrangement of hydroxyl groups on the cellulose chains, and results in "peeling" the chain from the surface [7] (Fig. A2.3).

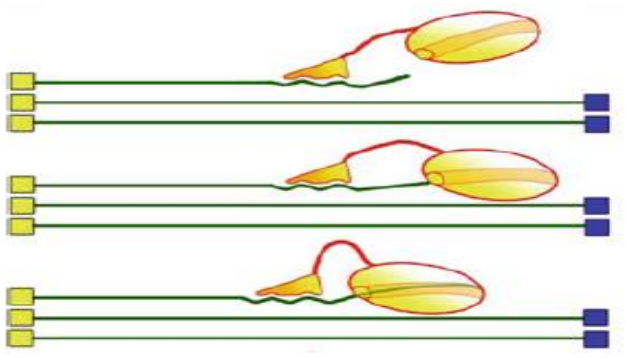

Figure A2.3 Action of CBH on cellulose chain (Figure 3 from Wang et al [7])

Action of enzymes was modeled accordingly in the model. During hydrolysis simulations, for each iteration location of bond inside a microfibril was determined corresponding to randomly chosen glucose molecule (by generating random number with uniform probability distribution out of a group of glucose molecules) to check its properties for specific enzyme action. To enable CBM binding (enzyme adsorption), a minimum number of glucose molecules are required on the elementary fibril surface and should not be blocked by other enzyme during that iteration. For endoglucanases, numbers of molecules were equal to size of enzymes (Fig. A2.2). In case of CBH, as the cellulose chain is peeled from the surface, it was assumed that glucose molecules equal to size of CBM only are required on surface and unblocked for binding. As the hydrolysis progress, soluble oligomers (chain length less than 7) get removed from the surface of the cellulose and part of chain just beneath the soluble chain is exposed and becomes accessible to enzymes.

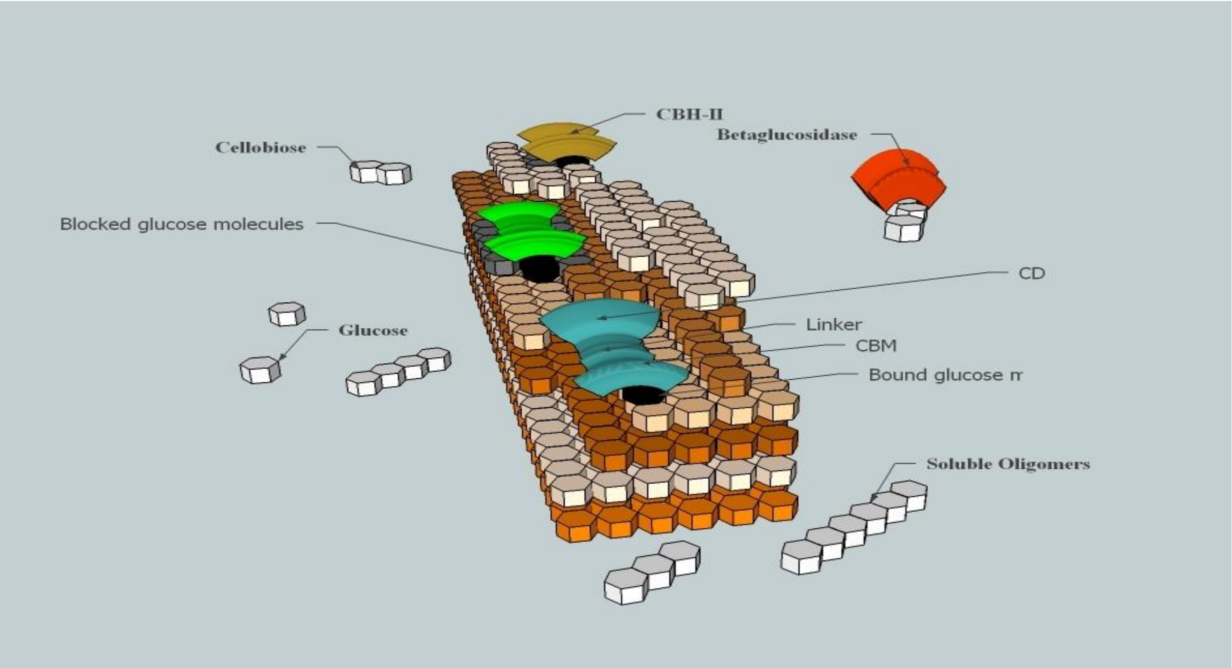

Figure A2.4 Action pattern and blocking of glucose molecules by enzymes simulated in the model

References

1. Mansfield SD, Mooney C, Saddler JN: **Substrate and enzyme characteristics that limit cellulose hydrolysis.** *Biotechnology progress* 1999, **15:**804-816.
2. Zhang YHP, Lynd LR: **Toward an aggregated understanding of enzymatic hydrolysis of cellulose: noncomplexed cellulase systems.** *Biotechnology and Bioengineering* 2004, **88:**797-824.
3. Levine SE, Fox JM, Blanch HW, Clark DS: **A mechanistic model of the enzymatic hydrolysis of cellulose.** *Biotechnology and Bioengineering* 2010, **107:**37-51.
4. Lynd LR, Weimer PJ, Van Zyl WH, Pretorius IS: **Microbial cellulose utilization: fundamentals and biotechnology.** *Microbiology and Molecular Biology Reviews* 2002, **66:**506-577.
5. Andersen N: **Enzymatic hydrolysis of cellulose—Experimental and modelling studies.** Technical University of Denmark, BioCentrum; 2007.
6. Chundawat SPS, Beckham GT, Himmel ME, Dale BE: **Deconstruction of lignocellulosic biomass to fuels and chemicals.** *Annual Review of Chemical and Biomolecular Engineering* 2011, **2:**121-145.
7. Wang M, Li Z, Fang X, Wang L, Qu Y: **Cellulolytic Enzyme Production and Enzymatic Hydrolysis for Second-Generation Bioethanol Production.** *Advances in Biochemical Engineering/Biotechnology* 2012**:**1-24.

Section A3: Values of parameters used for EG I, CBH I and CBH II action

The values of various parameters used in model simulations of cellulose hydrolysis by action of EG I, CBH I and CBH II are given in table below.

Parameters	EG I	CBH I	CBH II
Activity (IU/ mg protein)	0.4	0.8	0.16
Prob_EF_bind * (%)	75	75	75
N_{nb} **	0.2	0.005	0.005
N_{nP} $^{\chi}$	0.3	0.05	0.05
N_{inhib_G2} $^{\psi}$	0.3	0.5	0.5
N_{inhib_G} $^{\varphi}$	0.015	0.025	0.025

* Probability of binding on bonds that are on elementary fibril surface but not on microfibril surface
** Increment in the counter during hydrolysis simulation if bindings conditions were not met
$^{\chi}$ Increment in the counter during hydrolysis simulation when binding occurred but hydrolysis conditions were not met
$^{\psi}$ Increment in the counter during hydrolysis simulation when randomly chosen bond is a cellobiose molecule
$^{\varphi}$ Increment in the counter during hydrolysis simulation when randomly chosen bond is a glucose molecule

Section A4: Calculations of concentrations of soluble and insoluble sugars

Sugar concentrations were calculated at various time intervals to predict the hydrolysis profile during model simulations. Concentrations of different soluble (glucose, cellobiose, cellotriose, cellotetrose, cellopentose, cellohexose) and insoluble sugars were calculated using following equations (equations A4.1-A4.7):

$$C_{Glu} = \frac{N_{Glu}*G_{actual}}{G_{sim}} * \frac{180}{6.023*10^{23}} * \frac{1000}{V_{actual}} \quad \text{(A4.1)}$$

$$C_{G2} = \frac{N_{G2}*G_{actual}}{G_{sim}} * \frac{342}{6.023*10^{23}} * \frac{1000}{V_{actual}} \quad \text{(A4.2)}$$

$$C_{G3} = \frac{N_{G3}*G_{actual}}{G_{sim}} * \frac{504}{6.023*10^{23}} * \frac{1000}{V_{actual}} \quad \text{(A4.3)}$$

$$C_{G4} = \frac{N_{G4}*G_{actual}}{G_{sim}} * \frac{666}{6.023*10^{23}} * \frac{1000}{V_{actual}} \quad \text{(A4.4)}$$

$$C_{G5} = \frac{N_{G5}*G_{actual}}{G_{sim}} * \frac{828}{6.023*10^{23}} * \frac{1000}{V_{actual}} \quad \text{(A4.5)}$$

$$C_{G6} = \frac{N_{G6}*G_{actual}}{G_{sim}} * \frac{990}{6.023*10^{23}} * \frac{1000}{V_{actual}} \quad \text{(A4.6)}$$

$$C_{G6+} = \frac{N_{G6+}*G_{actual}}{G_{sim}} * \frac{162}{6.023*10^{23}} * \frac{1000}{V_{actual}} \quad \text{(A4.7)}$$

Where,

'C_{Glu}', 'C_{G2}', 'C_{G3}', 'C_{G4}', 'C_{G5}', 'C_{G6}', 'C_{G6+}' are concentrations of glucose, cellobiose, cellotriose, cellotetrose, cellopentose, cellohexose and high DP molecules in gram/L respectively. 'G_{actual}' is number of glucose molecules in actual sample (experimental conditions). 'G_{sim}' is number of glucose molecules simulated in the model. 'V_{actual}' is volume of solution in mL.

Number of glucose molecules in chain length greater than six ('N_{G6+}') and in actual sample ('G_{actual}') were calculated using equations A4.8 and A4.9 respectively.

$$N_{G6+} = G_{sim} - (N_{Glu} + 2 * N_{G2} + 3 * N_{G3} + 4 * N_{G4} + 5 * N_{G5} + 6 * N_{G6}) \quad \text{(A4.8)}$$

$$G_{actual} = \frac{W_{sample}*S*C* 6.023*10^{23}}{162} \quad \text{(A4.9)}$$

Where, 'W_{sample}' is weight of total solution during hydrolysis in grams. 'S' is fraction of solids in the solution (biomass loading), dimensionless. 'C' is cellulose fraction of the solid, dimensionless.

Section A5: Cellobiose production during hydrolysis of Avicel by CBH I action

This section presents the data from model simulation from hydrolysis of Avicel at various enzyme:substrate ratios. Simulations were performed three times at each condition and standard deviations are provided in the tables (A5.1 – A5.6) below.

Table A5.1 Cellobiose production during hydrolysis of Avicel (25 g/L) at CBHI loading of 4 mg/g glucans

	Cellobiose (g/L)					
Time (h)	Replication 1	Replication 2	Replication 3	Average	±	SD
0	0.0000	0.0000	0.0000	0.0000	±	0.0000
1	0.1234	0.1229	0.1181	0.1215	±	0.0030
2	0.2113	0.2110	0.2093	0.2105	±	0.0011
3	0.2829	0.2777	0.2696	0.2767	±	0.0067
4	0.3356	0.3338	0.3251	0.3315	±	0.0056
5	0.3757	0.3717	0.3723	0.3732	±	0.0022
6	0.4080	0.4164	0.4140	0.4128	±	0.0043
7	0.4529	0.4503	0.4540	0.4524	±	0.0019
8	0.4897	0.4878	0.4841	0.4872	±	0.0028
9	0.5189	0.5149	0.5132	0.5157	±	0.0029
10	0.5469	0.5469	0.5347	0.5429	±	0.0070
11	0.5785	0.5717	0.5598	0.5700	±	0.0095
12	0.5996	0.5970	0.5823	0.5930	±	0.0093
13	0.6323	0.6253	0.6102	0.6226	±	0.0113
14	0.6605	0.6513	0.6292	0.6470	±	0.0161
15	0.6844	0.6814	0.6536	0.6731	±	0.0170
16	0.7092	0.7025	0.6854	0.6990	±	0.0123
17	0.7355	0.7314	0.7056	0.7241	±	0.0162
18	0.7536	0.7536	0.7283	0.7451	±	0.0146
19	0.7795	0.7736	0.7545	0.7692	±	0.0131
20	0.7969	0.7969	0.7797	0.7912	±	0.0099
21	0.8170	0.8165	0.8005	0.8113	±	0.0094
22	0.8374	0.8377	0.8192	0.8315	±	0.0106
23	0.8547	0.8617	0.8336	0.8500	±	0.0146
24	0.8738	0.8808	0.8500	0.8682	±	0.0162
25	0.8867	0.8976	0.8725	0.8856	±	0.0126
26	0.9026	0.9130	0.8927	0.9028	±	0.0101

27	0.9187	0.9295	0.9135	0.9206	±	0.0082
28	0.9358	0.9492	0.9371	0.9407	±	0.0074
29	0.9555	0.9633	0.9559	0.9582	±	0.0044
30	0.9776	0.9879	0.9757	0.9804	±	0.0066
31	0.9974	1.0026	0.9935	0.9978	±	0.0046
32	1.0173	1.0167	1.0103	1.0148	±	0.0039
33	1.0341	1.0358	1.0200	1.0300	±	0.0087
34	1.0454	1.0475	1.0326	1.0418	±	0.0081
35	1.0676	1.0588	1.0497	1.0587	±	0.0090
36	1.0831	1.0763	1.0682	1.0759	±	0.0075
37	1.0976	1.0973	1.0860	1.0936	±	0.0066
38	1.1112	1.1088	1.1000	1.1067	±	0.0059
39	1.1288	1.1200	1.1139	1.1209	±	0.0075
40	1.1502	1.1335	1.1282	1.1373	±	0.0115
41	1.1615	1.1480	1.1480	1.1525	±	0.0078
42	1.1772	1.1628	1.1582	1.1661	±	0.0099
43	1.1886	1.1769	1.1724	1.1793	±	0.0083
44	1.2047	1.1898	1.1878	1.1941	±	0.0092
45	1.2205	1.2047	1.2051	1.2101	±	0.0090
46	1.2324	1.2197	1.2218	1.2246	±	0.0068
47	1.2440	1.2324	1.2344	1.2369	±	0.0062
48	1.2622	1.2439	1.2448	1.2503	±	0.0103

Table A5.2 Cellobiose production during hydrolysis of Avicel (25 g/L) at CBHI loading of 16.7 mg/g glucans

	Cellobiose (g/L)					
Time	Replication 1	Replication 2	Replication 3	Average	±	SD
0	0.0000	0.0000	0.0000	0.0000	±	0.0000
1	0.5191	0.5326	0.5243	0.5253	±	0.0068
2	0.8664	0.8624	0.8701	0.8663	±	0.0039
3	1.1233	1.0991	1.1302	1.1175	±	0.0163
4	1.3391	1.3084	1.3494	1.3323	±	0.0213
5	1.5188	1.4868	1.5272	1.5110	±	0.0213
6	1.6689	1.6317	1.6811	1.6606	±	0.0257
7	1.8190	1.7803	1.8331	1.8108	±	0.0273
8	1.9524	1.9328	1.9492	1.9448	±	0.0105
9	2.0866	2.0710	2.0829	2.0802	±	0.0082
10	2.1829	2.1725	2.1893	2.1816	±	0.0085

11	2.2650	2.3079	2.2906	2.2878 ± 0.0216
12	2.3540	2.4008	2.3901	2.3816 ± 0.0245
13	2.4392	2.4831	2.4813	2.4679 ± 0.0248
14	2.5361	2.5638	2.5587	2.5529 ± 0.0148
15	2.6041	2.6354	2.6357	2.6251 ± 0.0182
16	2.6855	2.7182	2.7308	2.7115 ± 0.0234
17	2.7662	2.7869	2.8147	2.7892 ± 0.0244
18	2.8277	2.8458	2.8805	2.8514 ± 0.0269
19	2.9038	2.9141	2.9506	2.9229 ± 0.0246
20	2.9771	2.9760	3.0261	2.9930 ± 0.0286
21	3.0267	3.0449	3.0943	3.0553 ± 0.0350
22	3.0845	3.0860	3.1597	3.1101 ± 0.0430
23	3.1321	3.1487	3.2142	3.1650 ± 0.0434
24	3.1904	3.2051	3.2669	3.2208 ± 0.0406
25	3.2430	3.2541	3.3068	3.2680 ± 0.0341
26	3.2871	3.2989	3.3540	3.3133 ± 0.0357
27	3.3306	3.3388	3.3991	3.3562 ± 0.0374
28	3.3831	3.3880	3.4487	3.4066 ± 0.0365
29	3.4303	3.4424	3.4865	3.4531 ± 0.0296
30	3.4796	3.4804	3.5273	3.4958 ± 0.0273
31	3.5171	3.5223	3.5676	3.5357 ± 0.0278
32	3.5732	3.5787	3.6090	3.5870 ± 0.0192
33	3.6228	3.6124	3.6445	3.6266 ± 0.0163
34	3.6652	3.6538	3.6892	3.6694 ± 0.0181
35	3.6980	3.6965	3.7214	3.7053 ± 0.0140
36	3.7376	3.7355	3.7609	3.7447 ± 0.0141
37	3.7798	3.7761	3.7933	3.7830 ± 0.0090
38	3.8187	3.8155	3.8263	3.8202 ± 0.0055
39	3.8451	3.8505	3.8591	3.8516 ± 0.0070
40	3.8763	3.8782	3.8977	3.8841 ± 0.0118
41	3.9136	3.9123	3.9261	3.9173 ± 0.0076
42	3.9411	3.9346	3.9570	3.9442 ± 0.0115
43	3.9667	3.9585	3.9863	3.9705 ± 0.0143
44	4.0052	3.9934	4.0174	4.0053 ± 0.0120
45	4.0389	4.0149	4.0509	4.0349 ± 0.0183
46	4.0564	4.0384	4.0787	4.0579 ± 0.0202
47	4.0879	4.0623	4.1053	4.0852 ± 0.0217
48	4.1071	4.0909	4.1336	4.1106 ± 0.0216

Table A5.3 Cellobiose production during hydrolysis of Avicel (25 g/L) at CBHI loading of 40 mg/g glucans

	Cellobiose (g/L)					
Time	Replication 1	Replication 2	Replication 3	Average	±	SD
0	0.0000	0.0000	0.0000	0.0000	±	0.0000
1	1.1104	1.0574	1.0829	1.0836	±	0.0265
2	1.6985	1.6731	1.7054	1.6923	±	0.0170
3	2.1632	2.1215	2.1524	2.1457	±	0.0216
4	2.5209	2.4959	2.5236	2.5135	±	0.0153
5	2.8337	2.8005	2.8355	2.8232	±	0.0197
6	3.0506	3.0547	3.0817	3.0623	±	0.0169
7	3.2586	3.2768	3.2993	3.2782	±	0.0204
8	3.4632	3.4786	3.4827	3.4748	±	0.0103
9	3.6453	3.6534	3.6543	3.6510	±	0.0050
10	3.7779	3.8308	3.7695	3.7927	±	0.0332
11	3.9143	3.9751	3.8955	3.9283	±	0.0416
12	4.0457	4.0768	4.0191	4.0472	±	0.0289
13	4.1499	4.1887	4.1210	4.1532	±	0.0340
14	4.2601	4.2839	4.2271	4.2571	±	0.0285
15	4.3710	4.3713	4.3294	4.3572	±	0.0241
16	4.4629	4.4591	4.4239	4.4486	±	0.0215
17	4.5546	4.5348	4.5164	4.5352	±	0.0191
18	4.6394	4.6165	4.6152	4.6237	±	0.0136
19	4.7076	4.6928	4.6902	4.6969	±	0.0094
20	4.7661	4.7572	4.7573	4.7602	±	0.0051
21	4.8312	4.8180	4.8157	4.8217	±	0.0084
22	4.9008	4.8854	4.8839	4.8900	±	0.0094
23	4.9619	4.9524	4.9298	4.9481	±	0.0165
24	5.0154	5.0054	4.9922	5.0043	±	0.0117
25	5.0768	5.0682	5.0417	5.0622	±	0.0183
26	5.1283	5.1286	5.0918	5.1162	±	0.0212
27	5.1737	5.1803	5.1393	5.1644	±	0.0220
28	5.2261	5.2374	5.1807	5.2147	±	0.0300
29	5.2807	5.2846	5.2279	5.2644	±	0.0317
30	5.3297	5.3344	5.2718	5.3119	±	0.0348
31	5.3722	5.3735	5.3123	5.3527	±	0.0350
32	5.4189	5.4258	5.3488	5.3978	±	0.0426
33	5.4507	5.4612	5.3873	5.4331	±	0.0400
34	5.4954	5.5028	5.4274	5.4752	±	0.0416

35	5.5384	5.5448	5.4714	5.5182	±	0.0407
36	5.5774	5.5839	5.5148	5.5587	±	0.0382
37	5.6109	5.6237	5.5492	5.5946	±	0.0399
38	5.6541	5.6722	5.5914	5.6392	±	0.0424
39	5.6853	5.7132	5.6359	5.6781	±	0.0392
40	5.7257	5.7518	5.6793	5.7189	±	0.0367
41	5.7621	5.7929	5.7148	5.7566	±	0.0394
42	5.8006	5.8330	5.7495	5.7944	±	0.0421
43	5.8400	5.8581	5.7792	5.8258	±	0.0413
44	5.8774	5.8941	5.8134	5.8616	±	0.0426
45	5.9145	5.9293	5.8480	5.8972	±	0.0433
46	5.9500	5.9630	5.8877	5.9336	±	0.0402
47	5.9906	5.9953	5.9222	5.9694	±	0.0409
48	6.0256	6.0424	5.9639	6.0106	±	0.0413

Table A5.4 Cellobiose production during hydrolysis of Avicel (50 g/L) at CBHI loading of 2 mg/g glucans

	Cellobiose (g/L)					
Time (h)	Replication 1	Replication 2	Replication 3	Average	±	SD
0	0.0000	0.0000	0.0000	0.0000	±	0.0000
1	0.0875	0.0929	0.0918	0.0907	±	0.0028
2	0.1525	0.1588	0.1572	0.1562	±	0.0033
3	0.2200	0.2125	0.2064	0.2130	±	0.0068
4	0.2746	0.2718	0.2506	0.2656	±	0.0131
5	0.3179	0.3145	0.2918	0.3080	±	0.0142
6	0.3523	0.3510	0.3340	0.3458	±	0.0102
7	0.3817	0.3920	0.3622	0.3786	±	0.0151
8	0.4116	0.4197	0.3966	0.4093	±	0.0117
9	0.4388	0.4472	0.4242	0.4367	±	0.0117
10	0.4644	0.4805	0.4558	0.4669	±	0.0125
11	0.4984	0.5052	0.4852	0.4963	±	0.0102
12	0.5235	0.5264	0.5172	0.5224	±	0.0047
13	0.5528	0.5560	0.5454	0.5514	±	0.0055
14	0.5792	0.5799	0.5646	0.5745	±	0.0086
15	0.6025	0.6041	0.5828	0.5964	±	0.0119
16	0.6276	0.6280	0.6072	0.6209	±	0.0119
17	0.6491	0.6528	0.6254	0.6424	±	0.0149
18	0.6635	0.6737	0.6490	0.6621	±	0.0124
19	0.6862	0.6905	0.6727	0.6831	±	0.0093
20	0.7053	0.7064	0.6929	0.7016	±	0.0075
21	0.7242	0.7219	0.7146	0.7202	±	0.0050

22	0.7414	0.7390	0.7336	0.7380	±	0.0040
23	0.7582	0.7531	0.7539	0.7551	±	0.0027
24	0.7801	0.7676	0.7686	0.7721	±	0.0069
25	0.7983	0.7782	0.7838	0.7868	±	0.0104
26	0.8241	0.7949	0.8029	0.8073	±	0.0151
27	0.8489	0.8110	0.8177	0.8259	±	0.0202
28	0.8624	0.8319	0.8350	0.8431	±	0.0168
29	0.8821	0.8530	0.8577	0.8643	±	0.0156
30	0.9002	0.8740	0.8786	0.8843	±	0.0140
31	0.9196	0.8916	0.8965	0.9026	±	0.0150
32	0.9374	0.9084	0.9138	0.9199	±	0.0154
33	0.9605	0.9261	0.9281	0.9382	±	0.0193
34	0.9744	0.9421	0.9500	0.9555	±	0.0169
35	0.9916	0.9527	0.9619	0.9687	±	0.0203
36	1.0142	0.9659	0.9713	0.9838	±	0.0265
37	1.0300	0.9808	0.9821	0.9976	±	0.0280
38	1.0412	0.9948	0.9969	1.0110	±	0.0262
39	1.0626	1.0062	1.0122	1.0270	±	0.0310
40	1.0801	1.0191	1.0222	1.0405	±	0.0344
41	1.0914	1.0278	1.0336	1.0509	±	0.0351
42	1.1094	1.0416	1.0496	1.0669	±	0.0370
43	1.1244	1.0513	1.0672	1.0809	±	0.0384
44	1.1410	1.0635	1.0814	1.0953	±	0.0406
45	1.1525	1.0725	1.0931	1.1060	±	0.0415
46	1.1695	1.0866	1.1083	1.1215	±	0.0430
47	1.1876	1.1035	1.1258	1.1390	±	0.0436
48	1.2082	1.1158	1.1340	1.1526	±	0.0490

Table A5.5 Cellobiose production during hydrolysis of Avicel (50 g/L) at CBHI loading of 8.3 mg/g glucans

	Cellobiose (g/L)					
Time	Replication 1	Replication 2	Replication 3	Average	±	SD
0	0.0000	0.0000	0.0000	0.0000	±	0.0000
1	0.5555	0.5469	0.5589	0.5538	±	0.0062
2	0.9535	0.9226	0.9379	0.9380	±	0.0154
3	1.2280	1.1847	1.2261	1.2129	±	0.0245
4	1.4268	1.4287	1.4532	1.4362	±	0.0147
5	1.6317	1.6316	1.6893	1.6509	±	0.0333
6	1.8126	1.7970	1.8486	1.8194	±	0.0265
7	1.9676	1.9618	2.0082	1.9792	±	0.0253
8	2.1007	2.1183	2.1208	2.1133	±	0.0110
9	2.2366	2.2479	2.2659	2.2501	±	0.0148
10	2.3804	2.3709	2.3813	2.3775	±	0.0057

11	2.5015	2.4957	2.4898	2.4957	±	0.0059
12	2.6090	2.6267	2.6050	2.6136	±	0.0115
13	2.7236	2.7222	2.7202	2.7220	±	0.0017
14	2.8336	2.8016	2.8200	2.8184	±	0.0161
15	2.9093	2.9231	2.9024	2.9116	±	0.0105
16	3.0041	3.0233	2.9936	3.0070	±	0.0151
17	3.0810	3.1159	3.0794	3.0921	±	0.0206
18	3.1774	3.2122	3.1741	3.1879	±	0.0211
19	3.2832	3.2787	3.2672	3.2764	±	0.0082
20	3.3935	3.3549	3.3322	3.3602	±	0.0310
21	3.4689	3.4314	3.4128	3.4377	±	0.0286
22	3.5380	3.5196	3.4733	3.5103	±	0.0333
23	3.6158	3.5920	3.5464	3.5848	±	0.0353
24	3.6794	3.6745	3.6171	3.6570	±	0.0346
25	3.7522	3.7636	3.6769	3.7309	±	0.0471
26	3.8191	3.8358	3.7617	3.8055	±	0.0389
27	3.8695	3.8970	3.8451	3.8705	±	0.0260
28	3.9555	3.9634	3.9191	3.9460	±	0.0236
29	4.0285	4.0197	3.9891	4.0124	±	0.0207
30	4.0847	4.0669	4.0574	4.0697	±	0.0139
31	4.1573	4.1324	4.1268	4.1388	±	0.0162
32	4.2033	4.1960	4.1863	4.1952	±	0.0085
33	4.2596	4.2520	4.2485	4.2533	±	0.0057
34	4.3121	4.3117	4.3223	4.3154	±	0.0060
35	4.3623	4.3670	4.3735	4.3676	±	0.0056
36	4.4201	4.4328	4.4203	4.4244	±	0.0073
37	4.4653	4.4791	4.4900	4.4782	±	0.0124
38	4.5285	4.5404	4.5409	4.5366	±	0.0070
39	4.5867	4.5914	4.5931	4.5904	±	0.0033
40	4.6490	4.6376	4.6509	4.6458	±	0.0072
41	4.7010	4.7006	4.7063	4.7026	±	0.0032
42	4.7427	4.7512	4.7616	4.7518	±	0.0095
43	4.7799	4.8076	4.8245	4.8040	±	0.0225
44	4.8244	4.8467	4.8946	4.8552	±	0.0359
45	4.8923	4.9014	4.9505	4.9148	±	0.0313
46	4.9321	4.9520	4.9962	4.9601	±	0.0328
47	4.9818	5.0009	5.0298	5.0041	±	0.0242
48	5.0287	5.0430	5.0682	5.0467	±	0.0200

Table A5.6 Cellobiose production during hydrolysis of Avicel (50 g/L) at CBHI loading of 20 mg/g glucans

	Cellobiose (g/L)					
Time	Replication 1	Replication 2	Replication 3	Average	±	SD

0	0.0000	0.0000	0.0000	0.0000	±	0.0000
1	1.2456	1.2280	1.2381	1.2372	±	0.0088
2	2.0545	2.0171	2.0411	2.0375	±	0.0190
3	2.6262	2.6001	2.6625	2.6296	±	0.0313
4	3.1302	3.0343	3.1385	3.1010	±	0.0579
5	3.5644	3.4480	3.5448	3.5191	±	0.0624
6	3.8616	3.7685	3.9168	3.8490	±	0.0749
7	4.2189	4.1138	4.2231	4.1853	±	0.0619
8	4.4822	4.4369	4.5167	4.4786	±	0.0400
9	4.7418	4.6440	4.8109	4.7322	±	0.0839
10	5.0546	4.8693	5.0793	5.0010	±	0.1148
11	5.2691	5.0879	5.3158	5.2243	±	0.1204
12	5.4430	5.3235	5.4914	5.4193	±	0.0865
13	5.6692	5.5270	5.6752	5.6238	±	0.0839
14	5.8588	5.7656	5.8688	5.8311	±	0.0569
15	6.0215	5.9848	6.0399	6.0154	±	0.0280
16	6.1758	6.1403	6.1940	6.1700	±	0.0273
17	6.3230	6.3045	6.3705	6.3327	±	0.0340
18	6.4894	6.4646	6.5224	6.4921	±	0.0290
19	6.6289	6.6014	6.6574	6.6292	±	0.0280
20	6.7816	6.7217	6.7506	6.7513	±	0.0299
21	6.9123	6.8349	6.8673	6.8715	±	0.0388
22	7.0246	6.9374	7.0131	6.9917	±	0.0474
23	7.1368	7.0444	7.1312	7.1041	±	0.0518
24	7.2436	7.1426	7.2135	7.1999	±	0.0518
25	7.3374	7.2754	7.3273	7.3134	±	0.0333
26	7.4241	7.3915	7.4215	7.4124	±	0.0181
27	7.5430	7.4886	7.5237	7.5184	±	0.0275
28	7.6648	7.5782	7.6068	7.6166	±	0.0441
29	7.7725	7.6808	7.6924	7.7152	±	0.0499
30	7.8621	7.7952	7.7651	7.8074	±	0.0497
31	7.9365	7.8818	7.8628	7.8937	±	0.0383
32	7.9962	7.9672	7.9481	7.9705	±	0.0242
33	8.0762	8.0412	8.0299	8.0491	±	0.0241
34	8.1655	8.1228	8.1140	8.1341	±	0.0275
35	8.2392	8.2105	8.1810	8.2102	±	0.0291
36	8.3089	8.2885	8.2378	8.2784	±	0.0366
37	8.3980	8.3832	8.3166	8.3659	±	0.0434
38	8.4717	8.4597	8.3938	8.4417	±	0.0419
39	8.5379	8.5077	8.4414	8.4956	±	0.0494
40	8.5939	8.5786	8.4928	8.5551	±	0.0545
41	8.6665	8.6302	8.5594	8.6187	±	0.0545
42	8.7455	8.6938	8.6287	8.6893	±	0.0585
43	8.8128	8.7688	8.6806	8.7541	±	0.0673
44	8.8613	8.8359	8.7389	8.8120	±	0.0646

45	8.9088	8.8845	8.7887	8.8607	±	0.0635
46	8.9634	8.9362	8.8329	8.9108	±	0.0689
47	9.0180	8.9842	8.8708	8.9577	±	0.0771
48	9.0772	9.0266	8.9249	9.0096	±	0.0776

Section A6: Endoglucanases action on substrates with different crystallinity

The figure A6.1 illustrates the hydrolysis profile of Avicel (semi-crystalline cellulose, CrI 0.5-0.6) and Cotton cellulose (highly crystalline cellulose) by action of endoglucanases. After 48h of hydrolysis, conversion of cotton cellulose was found 42.8% lower than that of Avicel.

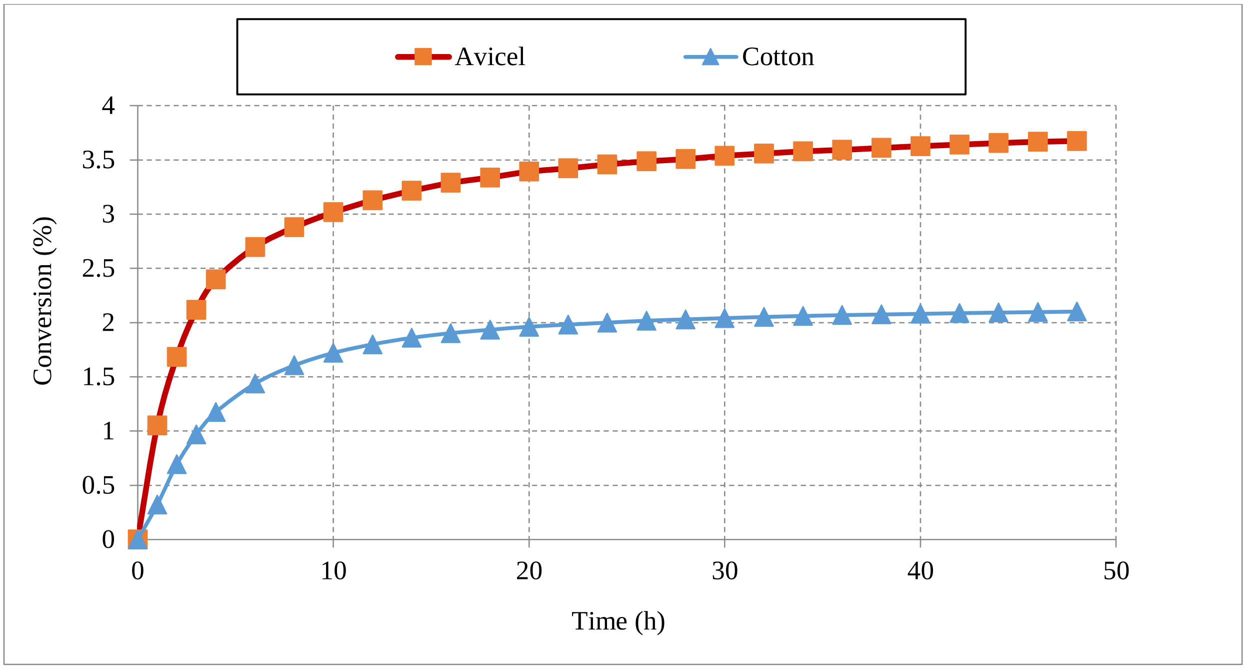

Figure A6.1 Model predictions: Action of endoglucanases (EG I) enzyme on Avicel and cotton cellulose.

Section A7: Effect of enzyme loading on the hydrolysis rate of cellulose

This section presents the data from model simulation from hydrolysis of Avicel at different loadings of enzyme mixture (12% EG I, 60% CBH I, 20% CBH II in presence of excess BG) (data used for figure 12).

Table A7.1 Effect of enzyme loading on cellulose hydrolysis rate

Hydrolysis Rate (g cellulose/h)			
Time (h)	Enzyme loading (mg enzyme/g glucan)		
	10 mg/g glucan	20 mg/g glucan	30 mg/g glucan
0	-	-	-
1	1.559	3.246	4.900
2	1.668	3.442	4.986
3	1.708	3.283	4.442
4	1.717	2.981	3.592
6	1.644	2.491	2.831
8	1.499	1.962	2.625
10	1.339	1.781	2.679
12	1.164	1.744	2.666
14	1.038	1.815	2.426
16	0.959	1.791	2.260
18	0.899	1.769	2.075
20	0.870	1.677	1.947
22	0.869	1.572	1.875
24	0.852	1.461	1.858
26	0.869	1.401	1.825
28	0.894	1.338	1.782
30	0.878	1.293	1.706
32	0.870	1.275	1.616
34	0.862	1.249	1.523
36	0.860	1.230	1.409
38	0.839	1.226	1.290
40	0.799	1.230	1.156
42	0.784	1.169	1.017
44	0.767	1.136	0.914
46	0.757	1.128	0.771
48	0.731	1.064	0.666
